# Blood Lipid Levels in Response to Almond Consumption: A Systematic Review and Meta-Analysis of Randomized Controlled Trials

**DOI:** 10.3390/nu17172791

**Published:** 2025-08-28

**Authors:** Kathy Musa-Veloso, Caroline Gauntlett, Katrina Geronimo, Isabella Vicente, Samuel Pak Lam Ho

**Affiliations:** Intertek Health Sciences Inc., 2233 Argentia Road, Suite 201, Mississauga, ON L5N 2X7, Canada; caroline.gauntlett@intertek.com (C.G.); katrina.geronimo@intertek.com (K.G.); isabella.vicente@intertek.com (I.V.); paklam.ho@intertek.com (S.P.L.H.)

**Keywords:** almonds, lipids, cholesterol, systematic review, meta-analysis

## Abstract

Background/Objectives: While the benefits of almond consumption in reducing levels of TC and LDL-C are well established, the effects on additional lipids that have emerged as important predictors of cardiovascular disease, such as ApoB and the ratio of ApoB:ApoA, are not well characterized. In this systematic review and meta-analysis, the effects of almond consumption on blood lipids were comprehensively assessed. Methods: On 12 May 2025, ProQuest Dialog™ was used to search ten literature databases (AdisInsight: Trials; Allied & Complementary Medicine™; BIOSIS Previews^®^; CAB ABSTRACTS; Embase^®^; Embase Preprints; Foodline^®^: SCIENCE; FSTA^®^; MEDLINE^®^; National Technical Information Service). Randomized controlled trials at least 4 weeks in duration were included if the investigational product was almonds; the control was void of nuts/tree nuts; the subjects were adults without CVD; and blood lipid levels were assessed. Health Canada’s Quality Appraisal Tool for Intervention Studies was used to assess each study’s risk of bias. The mean difference in the effect for each parameter was pooled across studies in a random effects model, using the inverse of the variance as the weighting factor. Results: 36 publications (48 almond–control datasets) representing 2485 participants were included. Almond consumption significantly reduced LDL-C (−0.132 mmol/L; 95% CI: −0.190, −0.075 mmol/L; *p* < 0.001), TC (−0.160 mmol/L; 95% CI: −0.218, −0.101 mmol/L; *p* < 0.001), non-HDL-C (−0.204 mmol/L; 95% CI: −0.281, −0.127 mmol/L; *p* < 0.001), TC:HDL-C (−0.154; 95% CI: −0.246, −0.063; *p* = 0.001), LDL-C:HDL-C (−0.112; 95% CI: −0.199, −0.026; *p* = 0.011), ApoB (−4.552 mg/dL; 95% CI: −6.460, −2.645 mg/dL; *p* < 0.001), and ApoB:ApoA (−0.027; 95% CI: −0.046, −0.008; *p* = 0.006), with a borderline significant reduction in TG (−0.037 mmol/L; 95% CI: −0.079, 0.005; *p* = 0.085) and no effects on HDL-C, ApoA, or Lp[a]. The effects persisted when the analyses were limited to higher quality studies, except for the reduction in TG. Conclusions: Almond consumption improves levels of LDL-C, TC, non-HDL-C, TC:HDL-C, LDL-C:HDL-C, ApoB, and ApoB:ApoA, though dedicated clinical trials are needed to better understand effects on TG levels.

## 1. Introduction

According to the World Health Organization (WHO), the leading cause of mortality globally is cardiovascular disease (CVD), with 32% of all global deaths in 2019 attributed to CVD [1]. While certain CVD risk factors are not modifiable (i.e., family history, older age, male gender, and certain ethnicities [African Americans, Hispanics, Latinos, and Southeast Asians]), other risk factors, including hypertension, diabetes, obesity, smoking, poor diet, sedentary lifestyle, and hyperlipidemia, are modifiable [2]. Hyperlipidemia is a major CVD risk factor, with elevations in atherogenic lipids contributing to atherosclerosis, an inflammatory disease characterized by fatty deposits in vessel walls, leading to their narrowing and hardening [3]. Atherosclerotic cardiovascular disease occurs when blood flow is compromised, leading to heart attacks, strokes, and peripheral arterial disease [3].

Hyperlipidemia is highly prevalent globally, as evidenced by the first report of the Global Diagnostics Network (GDN), which was published in 2023 by Martin et al. [4] and included a total of 461,888,753 lipid results (including total cholesterol [TC], low-density lipoprotein cholesterol [LDL-C], high-density lipoprotein cholesterol [HDL-C], and triglycerides [TG]) collected between 2018 and 2020 from adults aged 20 to 89 years residing in 17 countries on 5 different continents. An LDL-C cut-off of 3.36 mmol/L was met or exceeded in at least 20% of males and 20% of females in all countries except India [4]. Furthermore, the authors reported that the 5.0 mmol/L cut-off for TC established by the WHO as indicative of elevated CVD risk was exceeded in 7 of the 17 countries, including Japan, Australia, North Macedonia, Switzerland, Germany, Slovakia, and Austria [4]. As use of lipid-lowering medications was not an exclusion criterion in the GDN study, individuals presenting with normal levels of LDL-C and TC may have been pharmacologically treated with lipid-lowering agents, thereby underestimating the true prevalence of hyperlipidemia.

Dietary and lifestyle modifications that can improve blood lipid levels are important in reducing the risk of CVD. It has been demonstrated in several systematic reviews and meta-analyses that almond consumption improves levels of LDL-C and TC, though effects on TG and HDL-C have been less consistent [5,6,7]. The effects of almond consumption on levels of other blood lipids, including apolipoproteins A and B (ApoA and ApoB) and lipoprotein a (Lp[a]), have been investigated in the systematic review and meta-analysis by Lee-Bravatti et al. [6], who reported significant reductions in ApoB, with no effects on ApoA1 or Lp[a]. Lee-Bravatti et al. [6] did not assess the effects on non-HDL-C or on ApoB:ApoA; additionally, Lee-Bravatti et al. [6] conducted their literature search in 2017, and 8 years have since elapsed. Thus, a re-evaluation of the effects of almonds on the levels of the classic blood lipids (TC, LDL-C, HDL-C, and TG), as well as on levels of additional blood lipids that have emerged as important predictors of CVD risk (non-HDL-C, ApoA, ApoB, and Lp[a]) is needed. In the systematic review and meta-analysis presented herein, the effects of almonds on blood lipid levels are comprehensively assessed.

## 2. Methods

### 2.1. Literature Search

The present systematic review and meta-analysis was conducted in accordance with the guidelines of the Preferred Reporting Items for Systematic Reviews and Meta-Analyses (PRISMA) statement; the PRISMA checklist is provided in Supplementary Table S1 [8].

The literature search was conducted in duplicate by 2 reviewers working independently of one another using a similar methodology to that described by Musa-Veloso et al. [5]. Using the electronic search tool ProQuest Dialog™ (Version 75.0), 10 literature databases were searched on 12 May 2025 (AdisInsight: Trials, Allied & Complementary Medicine™, BIOSIS Previews^®^, CAB ABSTRACTS, Embase^®^, Embase Preprints, Foodline^®^: SCIENCE, FSTA^®^, MEDLINE^®^, and National Technical Information Service). Articles were identified if at least one exposure term (“almond”, “almonds”, “*Prunus amygdalus*”, “*P. amygdalus*”, “*Prunus dulcis*”, or “*P. dulcis*”) appeared in the title; at least one study population term (“men”, “women”, “man”, “woman”, “human”, “humans”, “subject”, “subjects”, “participant*”, “volunteer*”, “elder*”, “senior*”, “geriatric”, “adult*”, “teen*”, “adolescen*”, “people”, “person*”, “individual*”, or “patient*”) appeared in the title or abstract; and at least one study design term (“random*” or “control*”) appeared in the title or abstract. To restrict the search to studies conducted in humans, keywords related to pre-clinical studies (“animal*”, “rat”, “rats”, “mice”, “mouse”, “dog”, “dogs”, “pig”, “pigs”, “rabbit*”, “hamster*”, “monkey*”, “rodent*”, “in vitro”, or “ex vivo”) were required not to appear in the titles of the articles. No limitations pertaining to the health outcomes of interest, language, or year of publication were applied to the search. Following the automatic removal of duplicates by ProQuest Dialog™, the relevance of the publications was determined in duplicate at 3 stages using the titles, abstracts, and full-texts of publications. At each stage, inclusion/exclusion criteria were applied to determine literature relevance. To ensure the identification of all relevant studies, the reference lists of 6 relevant systematic reviews were cross-checked against the references identified in the literature search [6,7,9,10,11,12]. Where there were discrepancies in study inclusions and exclusions between the 2 reviewers, a 3rd reviewer was consulted, and a decision was reached through discussion and consensus.

### 2.2. Literature Filtration

To be eligible for inclusion, the study had to be a randomized controlled trial published as a full-length article in a peer-reviewed journal or as a publication pre-print or, if unpublished, the full study report had to be available; at least 5 study participants had to be enrolled per arm or group; study participants had to be adults (18 years of age or older, non-pregnant, and non-lactating) who were generally healthy or had elevated risk factors for CVD (e.g., diabetes, overweight/obesity, hyperlipidemia, family history of CVD) but did not have CVD; the control had to consist of either no food or any food(s), so long as the foods were not tree nuts/peanuts or fractions of tree nuts/peanuts; the amount of almonds administered had to be reported and had to be at least 5 g/day; the supplementation period had to be at least 4 weeks; the outcome of interest had to be related to fasting blood lipids (e.g., LDL-C, TC, HDL-C, non-HDL-C, TG, apolipoproteins, lipoproteins, or ratios of these) or risk of CVD; and the independent effects of almonds on blood lipid levels or risk of CVD had to be isolatable.

### 2.3. Data Extraction and Study Quality

Study data extraction and synthesis were conducted using the same methodology reported by Musa-Veloso et al. [5]. Specifically, data were extracted by 2 reviewers independently of one another, and the consistency of the 2 datasets was verified by a 3rd reviewer. If inconsistencies or discrepancies between the 2 datasets were identified, the original publication was consulted, and a consensus was reached via discussions between the 3 reviewers. Data extracted from the studies consisted of the study design; country of study conduct; initial and final sample sizes; study population (gender distribution, health status, and mean age); dietary interventions (dose, form of almonds, provision of foods or meals, and duration); mean blood lipid levels (i.e., LDL-C, TC, HDL-C, non-HDL-C, TC:HDL-C; LDL-C:HDL-C, TG, ApoA, ApoB, ApoB:ApoA, Lp[a]) at baseline and end-of-treatment; and the mean difference in the effect for each blood lipid parameter (see Data Synthesis section for details on the calculation of the mean difference in the effect for the crossover and parallel studies). For all studies, the end-of-treatment was defined as the day following the end of the control and almond intervention periods, regardless of the length of the supplementation period. A master spreadsheet was created with the references of each included study entered as a row heading and each lipid-related outcome as a column heading, thereby allowing for a detailed log of outcomes assessed across the studies and the subsequent grouping of studies by outcome.

Health Canada’s Quality Appraisal Tool for Intervention Studies [13] was used to assess study’s quality. Using this tool, a quantitative score (zero or one) was assigned to each of the 15 items included in the tool, and studies with scores of ≥8/15 were considered to be “higher quality”, while studies with scores of ≤7/15 were considered to be “lower quality”. A study with one or more of the following critical limitations was rated as “lower quality”, irrespective of the quality score:

- High attrition rate (>20%) and failure to conduct an intention-to-treat (ITT) analysis;
- Failure to report background dietary intakes;
- Significant differences between groups at baseline in energy, macronutrients, or fiber without statistical adjustments (e.g., analysis of covariance);
- Failure to report baseline lipid levels for each intervention group; or
- Significant differences between groups at baseline in lipid levels without statistical adjustments (e.g., analysis of covariance).

Study quality was appraised by 1 reviewer and verified by another reviewer. Where there were discrepancies in ratings, consensus was reached by deliberation and involvement of a 3rd reviewer.

### 2.4. Data Synthesis

The unit used for LDL-C, TC, HDL-C, non-HDL-C, and TG was mmol/L, while mg/dL was used for ApoA, ApoB, and Lp(a). If results were reported in units different from these, then conversion factors were applied to ensure consistency in the units. For each lipid-related outcome, the results of the studies were pooled in a meta-analysis, with the effect defined as the weighted mean difference, and the inverse of the variance used as the weighting factor. For parallel studies, the mean difference in the effect for each parameter was calculated as the change from baseline in the control group subtracted from the change from baseline in the almond group. For crossover studies, the mean difference in the effect for each parameter was calculated as the value at the end of the control phase subtracted from the value at the end of the almond phase. As the majority of studies in which the lipid-modifying effects of almonds were assessed did not report sufficient information for the calculation of the pre–post correlation, an assumed pre–post correlation of 0.5 was used for all studies, including studies in which computed effect sizes were reported, such that any error would affect all studies equally [14].

A random effects model was used, according to the methods described by DerSimonian and Laird [15], given that random effects models take into consideration the variability in response both within and between studies. For studies with multiple almond–control comparisons (hereinafter referred to as “strata”), each stratum was considered the unit of analysis only if the variable that was different between the strata was the dose of almonds; otherwise, the study was used as the unit of analysis. When each stratum was used as the unit of analysis, the sample size of the group that was shared across the strata was divided evenly amongst the comparisons so as to avoid a unit of analysis error (whereby the weight of each stratum is artificially inflated due to double-counting of subjects in the shared groups or arms) [16].

For the all the meta-analyses, sensitivity analyses were conducted for almond dose (<45 versus ≥45 g/day); baseline LDL-C level (<3.36 mmol/L [optimal] versus ≥3.36 mmol/L [not optimal]); study design (crossover versus parallel); study duration (<12 weeks versus ≥12 weeks); use of lipid-lowering medication (no use versus use by all or some subjects); provision of all meals and snacks to all subjects (provided versus not provided); and study quality (higher versus lower). If significant improvements in blood lipid levels were observed when analyses were limited to higher quality studies, then there was increased confidence in the certainty of the evidence. Of note, unlike the previous sensitivity analyses conducted by Musa-Veloso et al. [5], for baseline lipid status, the studies were segregated based only on LDL-C level at baseline so that the pools of studies remained consistent and effects on each lipid outcome in individuals below and above the LDL-C cut-off could be investigated. Additionally, unlike the previous sensitivity analysis conducted by Musa-Veloso et al. [5], in the present sensitivity analysis, 3.36 mmol/L was the cut-off used to define an LDL-C level that was and was not optimal, as an LDL-C value ≥ 3.36 mmol/L is categorized as borderline-high by the National Cholesterol Education Program in their Adult Treatment Panel III Guidelines [17].

The *I*^2^ statistic was used to evaluate between-study heterogeneity, with a value > 50% considered substantial [18]. When substantial heterogeneity was identified, studies were removed sequentially to determine whether the heterogeneity was related to any specific study. Publication bias was assessed using the Trim and Fill method described by Duval and Tweedie [19]. According to this method, if asymmetry in the funnel plot was determined to be the result of the presence of small studies (with large variances) in which large effect sizes were reported, with an unbalanced number of small studies showing a small effect, then those “missing” studies were imputed, and the pooled effect size was recalculated. Heterogeneity and publication bias were assessed only for the main analyses (not the sensitivity analyses); also, forest plots displaying individual study results and the overall weighted mean difference are provided only for the main analyses (not the sensitivity analyses). All analyses were conducted using Comprehensive Meta-analysis Software (version 4.0.000). For weighted mean differences, *p* < 0.05 was considered statistically significant, with 0.05 ≤ *p* < 0.10 considered borderline significant.

## 3. Results

### 3.1. Literature Search Results and Overview of Included Studies

**Figure 1:**
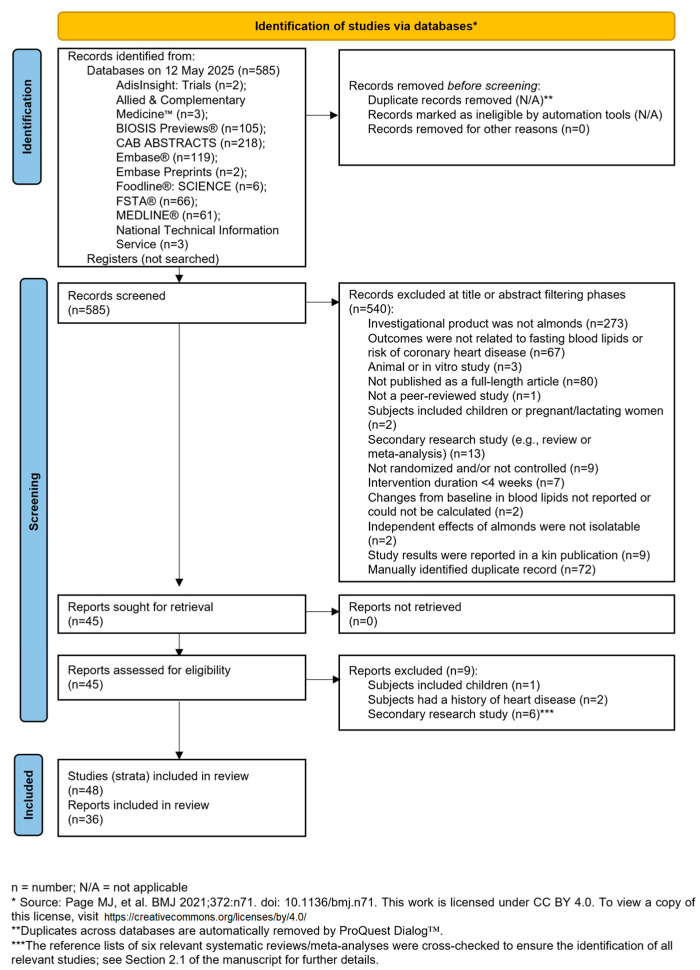
Flowchart of the literature search process [8].

The literature search process flowchart, constructed using the PRISMA template [8], is shown in Figure 1. The literature search resulted in the identification of 585 potentially relevant titles, of which 36 met all the inclusion criteria and none of the exclusion criteria [20,21,22,23,24,25,26,27,28,29,30,31,32,33,34,35,36,37,38,39,40,41,42,43,44,45,46,47,48,49,50,51,52,53,54,55]. Of note, no studies were excluded because the number of subjects per group was less than 5.

The key characteristics of the 36 included publications are summarized in Table 1. Across the 36 publications, the total number of subjects studied was 2485, with the number of study completers ranging from 6 to 178 per group in parallel studies and from 18 to 84 per arm in crossover studies. The 36 publications provided a total of 48 strata. Of the 48 strata, 30 were from parallel studies and 18 were from crossover studies. In 43 of the 48 strata, both males and females were included; of the 5 remaining strata, only females were studied in 3 strata [20] and [40] (strata 1 and 2), and only males were studied in 2 strata [38] (strata 1 and 2). The subjects were described in the publications as generally healthy in 4 strata [23,29], and [43] (strata 1 and 2); habitual smokers but without comorbidities in 2 strata [38] (strata 1 and 2); overweight or obese and without other comorbidities in 8 strata [20,31,36,39], [45] (strata 1 and 2) and [47,50] but with additional comorbidities in 8 strata [22,24,26,32,33], [41] (strata 1 and 2), and [46]; on stable statin therapy in 1 stratum [48]; having type 2 diabetes (T2D), prediabetes, or being at risk of T2D in 13 strata [25,27,34,35,42], [44] (strata 1 and 2), [52], [54] (strata 1 through 4) and [55]; having dyslipidemia in 11 strata [21,28], [37] (strata 1 and 2), [40] (strata 1 and 2), [49] (strata 1 and 2), [51] (strata 1 and 2), and [53]; and having above-average risk of developing CVD in 1 stratum [30].

In 33 of the 48 strata, none of the subjects were taking medications, while medication use by all or some of the subjects was reported in 15 of the strata. Notably, in 4 strata, all subjects had T2D, and all or the majority of subjects were taking a stable dose of oral hypoglycemic agents [27], [44] (strata 1 and 2) and [42]; although use of lipid-lowering medications was an exclusion criterion in 3 of these strata, in the study by Cohen and Johnston [27], approximately half of the subjects in the almond and control groups were taking a stable dose of lipid-lowering medications at the time of the study. In the study by Ruisinger et al. [48], all subjects were taking a stable dose of statins. In an additional 10 strata, some but not all subjects were taking medications for diet-related diseases, including hyperlipidemia [22,24,25,26,35], [37] (strata 1 and 2) and [46,52,55].

The baseline LDL-C level was considered optimal (<3.36 mmol/L) in 33 strata and not optimal (≥3.36 mmol/L) in 15 strata [20,21,28,30,33], [37] (strata 1 and 2), [41] (strata 1 and 2), [42], [49] (strata 1 and 2), [51] (strata 1 and 2), and [53].

### 3.2. Almond Interventions

Across all the studies, the average daily intake of almonds ranged from 25 to 168 g/day, and the duration of the almond consumption periods ranged from 4 to 72 weeks. Almonds were required to be consumed every day in all the studies except 4, in which subjects consumed 43 g of almonds 5 to 7 days per week [52], 28 g of almonds 5 days per week [27], 57 g of almonds 6 days per week [26], or 30 to 50 g of almonds 6 days per week [24].

Whole, raw (unblanched, unsalted) almonds were consumed in 18 strata [20,22,24,26,28,32,33,34,35], [37] (strata 1 and 2), [46,47,48,50], [51] (strata 1 and 2)] and [52]. In 5 strata [27] and [54] (strata 1 through 4), the almonds that were administered to the subjects were not specifically described as raw, unblanched almonds; however, based on the reported caloric value of the almonds, it was assumed that the almonds were most likely raw, unblanched almonds. Dry, roasted almonds were consumed in 5 strata [29,30,36,39,55]; a variety of almonds, namely whole (raw), roasted, and flavored almonds were administered in 1 stratum [31]; a choice of raw and/or roasted almonds was given in 1 stratum [23]; and almond powder was consumed in 1 stratum [53]. In 6 strata, the types of almonds administered (whole [raw] versus roasted) were not specified [40] (strata 1 and 2), [43] (strata 1 and 2), [45] (strata 1 and 2). In 11 strata, the almonds were provided to the subjects by incorporating the almonds into meals and snacks [21,25], [38] (strata 1 and 2), [41] (strata 1 and 2), [42], [44] (strata 1 and 2), and [49] (strata 1 and 2). The almonds administered were described in the studies by Berryman et al. [21], in which unsalted, whole, natural almonds with skins were provided to subjects as a snack; Li et al. [42] and Chen et al. [25], in which roasted, unsalted whole almonds with skins or derived powder were used in the preparation of meals and/or snacks; and Jia et al. [38] (strata 1 and 2), in which almond powder was used in the formulation of meals provided in the army unit canteen. In the remaining 6 strata in which all meals and snacks were provided, it was assumed that whole almonds, almond pieces, and ground almonds were used to prepare the meals [41] (strata 1 and 2), [44] (strata 1 and 2), and [49] (strata 1 and 2).

### 3.3. Control Foods and Diets

In 11 of the 48 strata, subjects were provided with all their meals and snacks, and the control and almond diets were matched for energy [41] (strata 1 and 2); energy and saturated fat (SFA) [21]; energy and protein [49] (strata 1 and 2); energy, protein, and SFA [42]; energy, protein, polyunsaturated fat (PUFA), and SFA [25]; and energy, carbohydrate, protein, monounsaturated fat (MUFA), PUFA, SFA, and total fat [44] (strata 1 and 2). Although the method through which the provided diets were matched in the study by Jia et al. [38] (strata 1 and 2) was not reported, it was assumed that the active and control diets were isocaloric, given that the subjects were provided with all their meals and snacks at an army canteen unit. In 22 of the 48 strata, the “control food” was prescribed (e.g., pretzels, cookies, muffins) and was matched for energy in 13 strata [22,23,24,26,29,30,35,39], [43] (strata 1 and 2), and [46,47,50]; energy and total fat in 1 stratum [28]; energy, total fat, and carbohydrate in 1 stratum [27]; energy, total fat, and protein in 3 strata [45] (stratum 1) and [51] (strata 1 and 2); carbohydrate in 1 stratum [45] (stratum 2); and energy, protein, PUFA, SFA, and fiber in 2 strata [37] (strata 1 and 2); macronutrient matching was not detailed for stratum 2 of Kurlandsky and Stote [40]. In 15 strata, a control food was not provided, and study subjects were instructed to consume their habitual diets [32,36,52], and [54] (strata 1 through 4) or to follow a specific healthy diet, such as a National Cholesterol Education Program diet [33,34], [40] (stratum 1), [48,53,55] or a low-calorie diet [20,31].

### 3.4. Study Quality

All 36 publications had a score of 8 or greater based on Health Canada’s Quality Appraisal Tool for Intervention Studies [13]; however, 7 of the publications (representing 10 strata) were downgraded to “lower quality” due to critical limitations. In the study by Liu et al. [43] (strata 1 and 2), there was a high attrition rate with no ITT analysis, and there were significant differences between groups in baseline LDL-C levels, though statistical adjustments (e.g., analysis of covariance) were not made. In the study by Gravesteijn et al. [33], there was a high attrition rate with no ITT analysis, and dietary intakes were not reported. In 3 additional studies, there was a high attrition rate with no ITT analysis [37] (strata 1 and 2), [41,46], and in 2 additional studies, background dietary intakes were not reported [20,31].

In the majority of the publications (24 of 36), 2 of 15 points were lost due to lack of blinding of subjects and outcome assessors (which is not unexpected, given that the investigational product was almonds or meals/snacks formulated with almonds). Of the 12 remaining publications, only that by Lovejoy et al. [44] (strata 1 and 2) was described as double-blinded; the other 11 publications were described as single-blinded, and each lost 1 of 15 points due to a lack of blinding of the subjects [22,23,24,26,28,29,30,41,43,45,53]. Across all 36 publications, the limitations that were identified included lack of reporting of allocation concealment (*n* = 31 publications); lack of reporting of randomization method, meaning the “appropriateness” of the randomization method, which also is a quality factor, could not be judged (*n* = 15 publications); results of an ITT analysis not provided (*n* = 26 publications); inclusion/exclusion criteria (for subject selection) not provided (*n* = 3 publications); potential confounders not considered *(*n = 3 publications); reasons for subject attrition not provided (*n* = 1 publication); and the methodology used to measure the health effect not provided *(*n = 2 publications). A detailed summary of the limitations identified across the studies is provided in Supplementary Table S2.

### 3.5. Effects of Almonds on Fasting Blood Lipids

A summary of the outcomes evaluated in each study is provided in Supplementary Table S3. The effects of almond consumption on TC and TG were assessed in all 48 strata, while the effects on LDL-C and HDL-C were assessed in 46 and 45 strata, respectively. For all of the other lipid-related outcomes, effects were reported in 9 to 18 strata. In Table 2 below, the pooled results, heterogeneity, and publication bias are presented for all outcomes. In Supplementary Table S4, the meta-analysis results, including the sensitivity analyses for the main analyses, are presented for all outcomes.

#### 3.5.1. LDL-C

**Figure 2:**
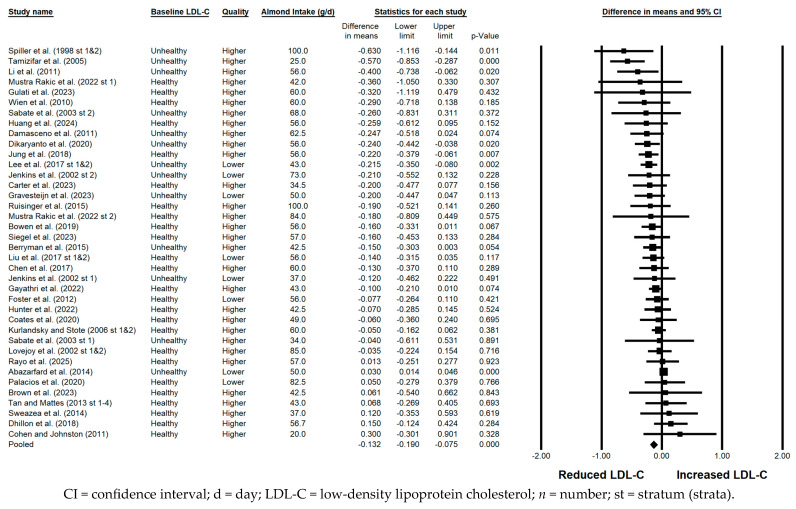
Effects of almonds on LDL-C across all studies (*n* = 35 publications and 46 strata) [20,21,22,23,24,25,26,27,28,29,30,31,32,33,34,35,36,37,39,40,41,42,43,44,45,46,47,48,49,50,51,52,53,54,55]. In this forest plot, for each study or stratum, a square symbol is used to represent the point estimate, bounded on either side by the 95% CI; the size of the square is proportional to the weight contributed by the study or stratum. A random effects model was used. With a mean almond intake of 20 to 100 g/d, the pooled change in LDL-C and associated 95% CI, represented by the diamond symbol, is −0.132 mmol/L (95% CI, −0.190, −0.075 mmol/L; *p* < 0.001).

LDL-C was significantly reduced with the consumption of almonds versus a control when the results from all 46 strata were pooled (−0.132 mmol/L; 95% confidence interval [CI]: −0.190, −0.075 mmol/L; *p* < 0.001; Figure 2). Publication bias was significant, with 5 studies found to be missing from the right of the pooled effect; with the imputation of these 5 studies, the reduction in LDL-C was −0.11 mmol/L (95% CI: −0.16, −0.05 mmol/L). Substantial heterogeneity was observed when all 46 strata were pooled; however, removal from the meta-analysis of the study by Abazarfard et al. [20] resulted in a reduction of the *I*^2^ statistic from 63.215% to 6.609% and a change in the associated *p*-value for heterogeneity from <0.001 to 0.355. The pooled reduction in LDL-C without the study by Abazarfard et al. [20] was −0.14 mmol/L (95% CI: −0.18, −0.10 mmol/L; *p* < 0.001). A significant reduction in LDL-C was observed in each of the sensitivity analyses except when the 10 lower quality strata were pooled, in which case the −0.101 mmol/L reduction in LDL-C approached significance (95% CI: −0.209, 0.008 mmol/L; *p* = 0.068). Of note, the magnitude of LDL-C lowering was similar, irrespective of almond dose (<45 g/day versus ≥45 g/day), use of lipid-lowering medication, or the provision of all meals and snacks. However, the reduction in LDL-C was greater when strata in which the baseline LDL-C level was not optimal were pooled (−0.229 mmol/L) compared to when strata in which the baseline LDL-C level was optimal were pooled (−0.094 mmol/L); when strata that were crossover in design were pooled (−0.179 mmol/L) compared to when strata that were parallel in design were pooled (−0.090 mmol/L); and when strata that were <12 weeks in duration were pooled (−0.156 mmol/L) compared to when strata that were ≥12 weeks in duration were pooled (−0.083 mmol/L).

#### 3.5.2. TC

**Figure 3:**
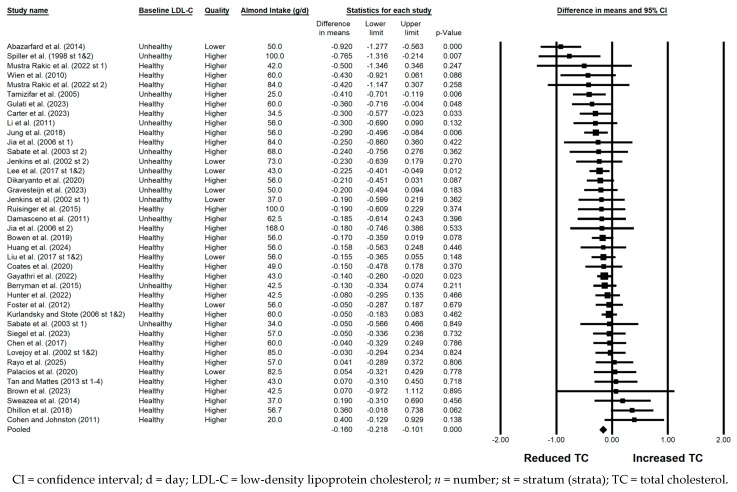
Effects of almonds on TC across all studies (*n* = 36 publications and 48 strata) [20,21,22,23,24,25,26,27,28,29,30,31,32,33,34,35,36,37,38,39,40,41,42,43,44,45,46,47,48,49,50,51,52,53,54,55]. In this forest plot, for each study or stratum, a square symbol is used to represent the point estimate, bounded on either side by the 95% CI; the size of the square is proportional to the weight contributed by the study or stratum. A random effects model was used. With a mean almond intake of 20 to 168 g/d, the pooled change in TC and associated 95% CI, represented by the diamond symbol, is –0.160 mmol/L (95% CI, −0.218, −0.101 mmol/L; *p* < 0.001).

TC was significantly reduced with the consumption of almonds versus a control when the results from all 48 strata were pooled (−0.160 mmol/L; 95% CI: −0.218, −0.101 mmol/L; *p* < 0.001; Figure 3). Publication bias was significant, with 3 studies found to be missing from the right of the pooled effect; with the imputation of these 3 studies, the reduction in TC was −0.13 mmol/L (95% CI: −0.20, −0.07 mmol/L). The *I*^2^ statistic was less than 50%, so heterogeneity was considered not substantial, despite being statistically significant. A significant reduction in TC was observed in each of the sensitivity analyses. Of note, the magnitude of TC lowering was similar, irrespective of almond dose (<45 g/day versus ≥45 g/day), study design, study duration, use of lipid-lowering medication, or the provision of all meals and snacks. However, the reduction in TC was greater when strata from lower quality studies were pooled (−0.228 mmol/L) compared to when strata from higher quality studies were pooled (−0.140 mmol/L), and when strata in which the baseline LDL-C level was not optimal were pooled (−0.290 mmol/L) compared to when strata in which the baseline LDL-C level was optimal were pooled (−0.109 mmol/L).

#### 3.5.3. HDL-C

**Figure 4:**
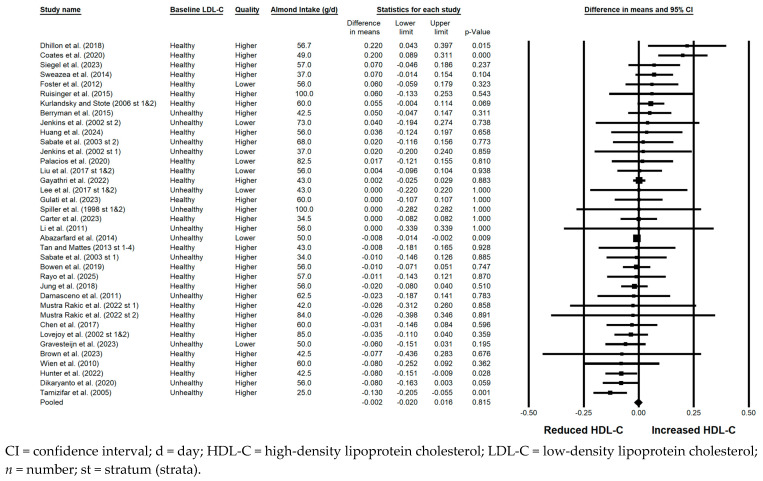
Effects of almonds on HDL-C across all studies (*n* = 34 publications and 45 strata) [20,21,22,23,24,25,26,28,29,30,31,32,33,34,35,36,37,39,40,41,42,43,44,45,46,47,48,49,50,51,52,53,54,55]. In this forest plot, for each study or stratum, a square symbol is used to represent the point estimate, bounded on either side by the 95% CI; the size of the square is proportional to the weight contributed by the study or stratum. A random effects model was used. With a mean almond intake of 25 to 100 g/d, the pooled change in HDL-C and associated 95% CI, represented by the diamond symbol, is −0.002 mmol/L (95% CI, −0.020, 0.016 mmol/L; *p* = 0.815).

There was no significant effect of almonds versus a control on HDL-C when the results from all 45 strata were pooled (−0.002 mmol/L; 95% CI: −0.020, 0.016 mmol/L; *p* = 0.815; Figure 4). Publication bias was not significant, as no studies were found to be missing to the left of the pooled effect. The *I*^2^ statistic was less than 50%, so heterogeneity was considered not substantial, despite being statistically significant. Among the sensitivity analyses, a very small but significant reduction in HDL-C was observed with the consumption of almonds versus a control only when strata from lower quality studies were pooled (−0.008 mmol/L; 95% CI: −0.014, −0.002 mmol/L; *p* = 0.009). Borderline significant reductions in HDL-C with the consumption of almonds versus a control were observed when strata in which the baseline LDL-C level was not optimal were pooled (−0.028 mmol/L; 95% CI: −0.060, 0.004 mmol/L; *p* = 0.083) and when strata from studies that were crossover in design were pooled (−0.025 mmol/L; 95% CI: −0.052, 0.003 mmol/L; *p* = 0.077).

#### 3.5.4. Non-HDL-C

**Figure 5:**
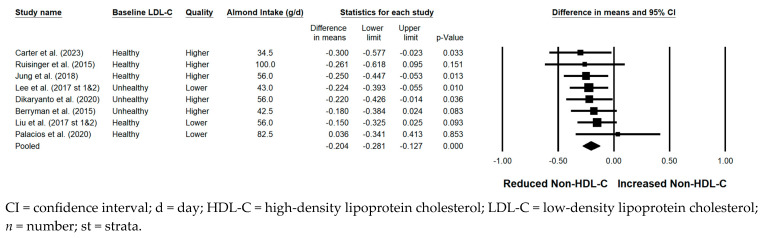
Effects of almonds on non-HDL-C across all studies (*n* = 8 publications and 10 strata) [21,24,30,39,41,43,46,48]. In this forest plot, for each study, a square symbol is used to represent the point estimate, bounded on either side by the 95% CI; the size of the square is proportional to the weight contributed by the study. A random effects model was used. With a mean almond intake of 34.5 to 100 g/d, the pooled change in non-HDL-C and associated 95% CI, represented by the diamond symbol, is −0.204 mmol/L (95% CI, −0.281, −0.127 mmol/L; *p* < 0.001).

Non-HDL-C was significantly reduced with the consumption of almonds versus a control when the results from all 10 strata were pooled (−0.204 mmol/L; 95% CI: −0.281, −0.127 mmol/L; *p* < 0.001; Figure 5). Publication bias was significant, with 1 study found to be missing from the right of the pooled effect; with the imputation of this one study, the reduction in non-HDL-C was −0.20 mmol/L (95% CI: −0.27, −0.12 mmol/L). The *I*^2^ statistic was 0%, indicating no heterogeneity. A significant reduction in non-HDL-C was observed in each of the sensitivity analyses. Of note, the magnitude of non-HDL-C lowering was similar, irrespective of almond dose (<45 g/day versus ≥45 g/day), baseline LDL-C level (<3.36 mmol/L versus ≥3.36 mmol/L), study design, study duration, use of lipid-lowering medication, or the provision of all meals and snacks. However, the reduction in non-HDL-C was greater when strata from higher quality studies were pooled (−0.232 mmol/L) compared to when strata from lower quality studies were pooled (−0.167 mmol/L).

#### 3.5.5. TC:HDL-C

**Figure 6:**
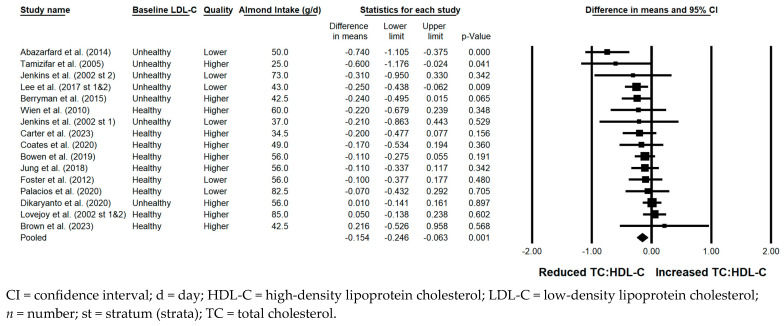
Effects of almonds on the ratio of TC:HDL-C across all studies (*n* = 15 publications and 18 strata) [20,21,22,23,24,26,30,31,37,39,41,44,46,53,55]. In this forest plot, for each study or stratum, a square symbol is used to represent the point estimate, bounded on either side by the 95% CI; the size of the square is proportional to the weight contributed by the study or stratum. A random effects model was used. With a mean almond intake of 25 to 85 g/d, the pooled change in the ratio of TC:HDL-C and associated 95% CI, represented by the diamond symbol, is −0.154 (95% CI, −0.246, −0.063; *p* = 0.001).

The TC:HDL-C ratio was significantly reduced with the consumption of almonds versus a control when the results from all 18 strata were pooled (−0.154; 95% CI: −0.246, −0.063; *p* = 0.001; Figure 6). Publication bias was significant, with 5 studies found to be missing from the right of the pooled effect; with the imputation of these 5 studies, the reduction in TC:HDL-C was −0.07 (95% CI: −0.18, 0.03). The *I*^2^ statistic was 38.129%, indicating no substantial heterogeneity. A significant reduction in TC:HDL-C was observed in each of the sensitivity analyses except when all 5 strata in which all meals and snacks were provided were pooled and when the 10 strata in which the baseline LDL-C was optimal were pooled, with the latter reduction approaching significance (−0.084; 95% CI: −0.171, 0.003; *p* = 0.057). The reduction in TC:HDL-C was similar, irrespective of study design, use of lipid-lowering medication, or the provision of all meals and snacks. However, the reduction in TC:HDL-C was greater when strata in which the baseline LDL-C level was not optimal were pooled (−0.291) compared to when strata in which the baseline LDL-C level was optimal were pooled (−0.084); when strata in which the almond dose was <45 g/day were pooled (−0.239) compared to when strata in which the almond dose was ≥45 g/day were pooled (−0.123); when strata from studies that were ≥12 weeks in duration were pooled (−0.242) compared to when strata from studies that were <12 weeks in duration were pooled (−0.110); and when strata from studies that were lower quality were pooled (−0.270) compared to when strata from studies that were higher quality were pooled (−0.088).

#### 3.5.6. LDL-C:HDL-C

**Figure 7:**
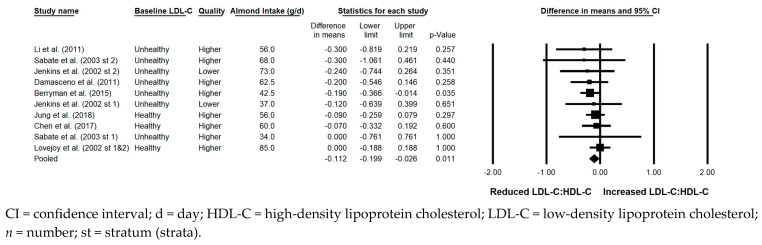
Effects of almonds on the ratio of LDL-C:HDL-C across all studies (*n* = 8 publications and 11 strata) [21,25,28,37,39,42,44,49]. In this forest plot, for each study or stratum, a square symbol is used to represent the point estimate, bounded on either side by the 95% CI; the size of the square is proportional to the weight contributed by the study or stratum. A random effects model was used. With a mean almond intake of 34 to 85 g/d, the pooled change in the ratio of LDL-C:HDL-C and associated 95% CI, represented by the diamond symbol, is −0.112 (95% CI, −0.199, −0.026; *p* = 0.011).

The LDL-C:HDL-C ratio was significantly reduced with the consumption of almonds versus a control when the results from all 11 strata were pooled (−0.112; 95% CI: −0.199, −0.026; *p* = 0.011; Figure 7). Publication bias was significant, with 2 studies found to be missing from the right of the pooled effect; with the imputation of these 2 studies, the reduction in LDL-C:HDL-C was −0.10 (95% CI: −0.19, −0.02). The *I*^2^ statistic was 0%, indicating no heterogeneity. A sensitivity analysis could not be conducted for the effect of study design, as all strata were from studies that were crossover in design; likewise, a sensitivity analysis could not be conducted for study duration, as there was only 1 stratum from a study ≥ 12 weeks in duration. Among the remaining sensitivity analyses, the reduction in LDL-C:HDL-C was significant or nearly significant, except for when strata in which the baseline LDL-C level was optimal were pooled; when strata in which some or all subjects were taking lipid-lowering medications were pooled; and when strata from lower quality studies were pooled. The reduction in LDL-C:HDL-C was similar, irrespective of the provision of all meals and snacks. However, the reduction in LDL-C:HDL-C was greater when strata in which the almond dose was <45 g/day were pooled (−0.174) compared to when strata in which the almond dose was ≥45 g/day were pooled (−0.088).

#### 3.5.7. TG

**Figure 8:**
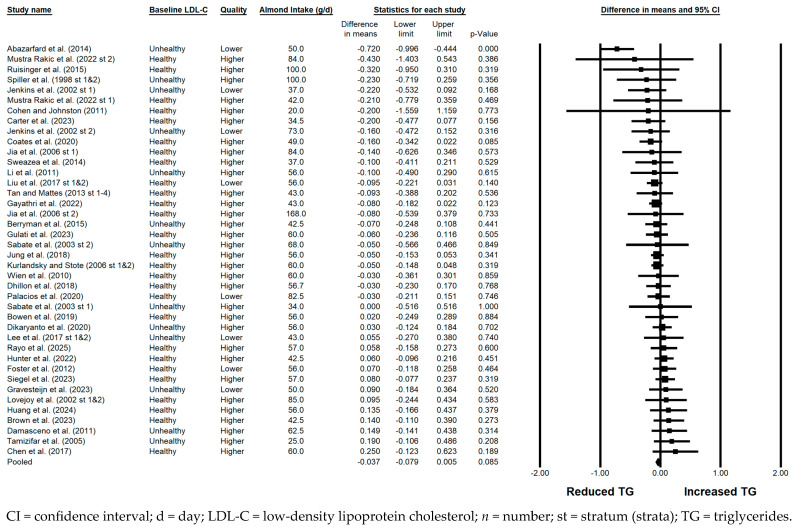
Effects of almonds on TG across all studies (*n* = 36 publications and 48 strata) [20,21,22,23,24,25,26,27,28,29,30,31,32,33,34,35,36,37,38,39,40,41,42,43,44,45,46,47,48,49,50,51,52,53,54,55]. In this forest plot, for each study or stratum, a square symbol is used to represent the point estimate, bounded on either side by the 95% CI; the size of the square is proportional to the weight contributed by the study or stratum. A random effects model was used. With a mean almond intake of 20 to 168 g/d, the pooled change in TG and associated 95% CI, represented by the diamond symbol, is −0.037 mmol/L (95% CI, −0.079, 0.005 mmol/L; *p* = 0.085).

There was a small but borderline significant reduction in TG with the consumption of almonds versus a control when all 48 strata were pooled (−0.037 mmol/L; 95% CI: −0.079, 0.005 mmol/L; *p* = 0.085; Figure 8). Publication bias was significant, with 3 studies found to be missing to the right of the pooled effect; with the imputation of these 3 studies, the reduction in TG was not significant (−0.02 mmol/L; 95% CI: −0.07, 0.03 mmol/L). The *I*^2^ statistic was 23.299%, for which heterogeneity was considered not substantial. Among the sensitivity analyses, a small but significant reduction in TG was observed with the consumption of almonds versus a control only when strata from studies that were parallel in design were pooled (−0.067 mmol/L; 95% CI: −0.127, −0.007 mmol/L; *p* = 0.028). Borderline significant reductions in TG with the consumption of almonds versus a control were observed when strata in which the baseline LDL-C level was optimal were pooled (−0.034 mmol/L; 95% CI: −0.071, 0.003 mmol/L; *p* = 0.075), when strata in which some or all subjects were using lipid-lowering medications were pooled (−0.073 mmol/L; 95% CI: −0.155, 0.009 mmol/L; *p* = 0.079), and when strata in which all meals and snacks were not provided were pooled (−0.042 mmol/L; 95% CI: −0.091, 0.007; *p* = 0.095).

#### 3.5.8. ApoA

**Figure 9:**
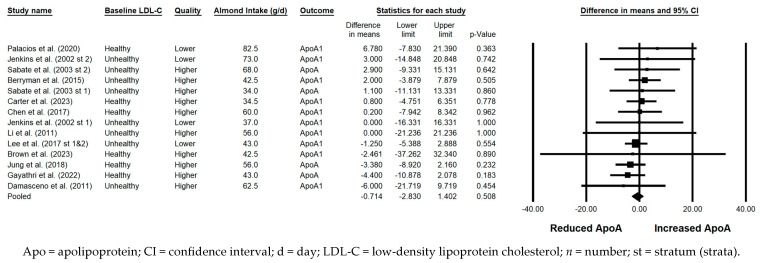
Effects of almonds on ApoA across all studies (*n* = 12 publications and 15 strata) [21,23,24,25,28,32,37,39,41,42,46,49] ^a^. In this forest plot, for each study or stratum, a square symbol is used to represent the point estimate, bounded on either side by the 95% CI; the size of the square is proportional to the weight contributed by the study or stratum. A random effects model was used. With a mean almond intake of 34 to 82.5 g/d, the pooled change in ApoA and associated 95% CI, represented by the diamond symbol, is −0.714 mg/dL (95% CI, −2.830, 1.402 mg/dL; *p* = 0.508). ^a^ For the studies that reported the results for ApoA1, the results for this outcome were combined with the results for ApoA in the meta-analysis.

There was no significant effect of almond consumption versus a control on ApoA when the results from 15 strata were pooled (−0.714 mg/dL; 95% CI: −2.830, 1.402 mg/dL; *p* = 0.508; Figure 9). Publication bias was significant, with 2 studies found to be missing to the left of the pooled effect; with the imputation of these 2 studies, the pooled effect for ApoA was −0.93 mg/dL (95% CI: −3.01, 1.15 mg/dL). The *I*^2^ statistic was 0%, indicating no heterogeneity. There were no significant effects of almond consumption versus a control on ApoA in any of the sensitivity analyses.

#### 3.5.9. ApoB

**Figure 10:**
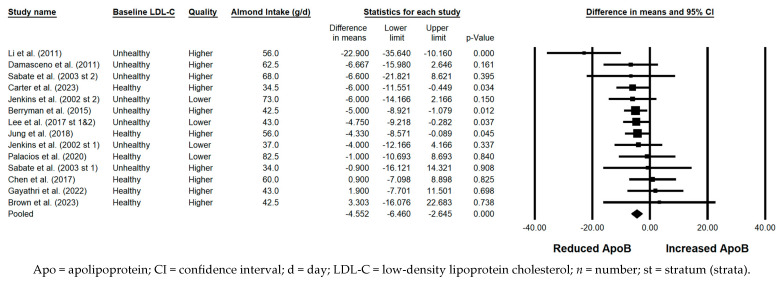
Effects of almonds on ApoB across all studies (*n* = 12 publications and 15 strata) [21,23,24,25,28,32,37,39,41,42,46,49]. In this forest plot, for each study or stratum, a square symbol is used to represent the point estimate, bounded on either side by the 95% CI; the size of the square is proportional to the weight contributed by the study or stratum. A random effects model was used. With a mean almond intake of 34 to 82.5 g/d, the pooled change in ApoB and associated 95% CI, represented by the diamond symbol, is −4.552 mg/dL (95% CI, −6.460, −2.645 mg/dL; *p* < 0.001).

ApoB was significantly reduced with the consumption of almonds versus a control when the results from all 15 strata were pooled (−4.552 mg/dL; 95% CI: −6.460, −2.645 mg/dL; *p* < 0.001; Figure 10). Publication bias was not significant, as no studies were found to be missing to the right of the pooled effect. The *I*^2^ statistic was 4.349%, indicating no substantial heterogeneity. A significant reduction in ApoB was observed in each of the sensitivity analyses except those limited to strata from studies that were parallel in design and those limited to strata with a duration ≥12 weeks. Of note, the magnitude of ApoB lowering was similar, irrespective of almond dose (<45 g/day versus ≥45 g/day), use of lipid-lowering medication, study quality, or the provision of all meals and snacks. However, the reduction in ApoB was greater when strata in which the baseline LDL-C level was not optimal were pooled (−5.776 mg/dL) compared to when strata in which the baseline LDL-C level was optimal were pooled (−3.149 mg/dL); when strata from studies that were crossover in design were pooled (−4.757 mg/dL) compared to when strata from studies that were parallel in design were pooled (−2.914 mg/dL); and when strata from studies that were shorter than 12 weeks in duration were pooled (–5.111 mg/dL) compared to when strata from studies that were ≥12 weeks were pooled (–2.145 mg/dL).

#### 3.5.10. ApoB:ApoA

**Figure 11:**
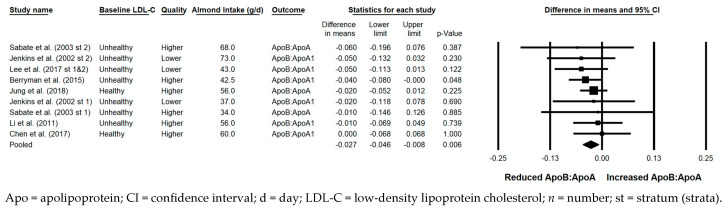
Effects of almonds on the ratio of ApoB:ApoA across all studies (*n* = 7 publications and 10 strata) [21,25,37,39,41,42,49] ^a^. In this forest plot, for each study or stratum, a square symbol is used to represent the point estimate, bounded on either side by the 95% CI; the size of the square is proportional to the weight contributed by the study or stratum. A random effects model was used. With a mean almond intake of 34 to 73 g/d, the pooled change in the ratio of ApoB:ApoA and associated 95% CI, represented by the diamond symbol, is −0.027 (95% CI, −0.046, −0.008; *p* = 0.006). ^a^ For the studies that reported the results for the ratio of ApoB:ApoA1, the results for this outcome were combined with the results for the ratio of ApoB:ApoA in the meta-analysis.

The ApoB:ApoA ratio was significantly reduced with the consumption of almonds versus a control when the results from all 10 strata were pooled (−0.027; 95% CI: −0.046, −0.008; *p* = 0.006; Figure 11). Publication bias was significant, with 1 study found to be missing to the right of the pooled effect; with the imputation of this study, the reduction in ApoA:ApoB was −0.03 (95% CI: −0.05, −0.01). The *I*^2^ statistic was 0%, indicating no heterogeneity. A sensitivity analysis could not be conducted for the effect of study design, as all strata were from studies that were crossover in design; likewise, a sensitivity analysis could not be conducted for study duration, as there was only one stratum that was from a study ≥12 weeks in duration. Among the remaining sensitivity analyses, the reduction in ApoB:ApoA was significant or nearly significant, except when strata in which the dose of almonds was ≥45 g/day were pooled, strata in which the baseline LDL-C was optimal were pooled, strata in which some or all subjects were taking lipid-lowering medications were pooled, and strata in which all meals and snacks were not provided were pooled.

#### 3.5.11. Lp(a)

**Figure 12:**
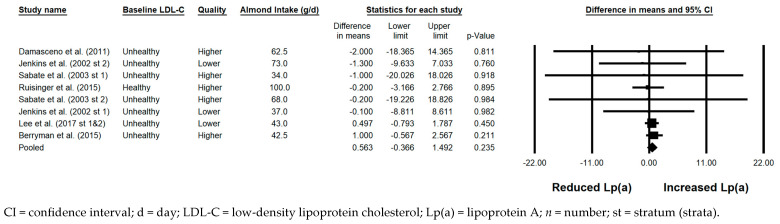
Effects of almonds on Lp(a) across all studies (*n* = 6 publications and 9 strata) [21,28,37,41,48,49]. In this forest plot, for each study or stratum, a square symbol is used to represent the point estimate, bounded on either side by the 95% CI; the size of the square is proportional to the weight contributed by the study or stratum. A random effects model was used. With a mean almond intake of 34 to 100 g/d, the pooled change in Lp(a) and associated 95% CI, represented by the diamond symbol, is 0.563 mg/dL (95% CI, −0.366, 1.492 mg/dL; *p* = 0.235).

There was no significant effect of almond consumption versus a control on Lp(a) when the results from all 9 strata were pooled (0.563 mg/dL; 95% CI: −0.366, 1.492 mg/dL; *p* = 0.235; Figure 12). Publication bias was significant, with 4 studies found to be missing to the right of the pooled effect; with the imputation of these 4 studies, the pooled effect for Lp(a) was 0.69 mg/dL (95% CI: −0.19, 1.57 mg/dL). The *I*^2^ statistic was 0%, indicating no heterogeneity. In none of the sensitivity analyses was there an effect of almond consumption versus a control on Lp(a), though there were insufficient data to examine the effects of almond consumption versus a control in studies in which the baseline LDL-C level was optimal, in studies with a parallel design, and in studies with a duration ≥12 weeks.

## 4. Discussion

Based on the analyses presented herein, almond consumption is associated with significant reductions in LDL-C, TC, non-HDL-C, the ratios of TC:HDL-C and LDL-C:HDL-C, ApoB, and the ratio of ApoB:ApoA, with a borderline significant reduction in TG, and no significant effects on HDL-C, ApoA, or Lp(a). These observations are largely consistent with those reported in previous systematic reviews and meta-analyses, though our finding of almond-associated reductions in the ratio of ApoB:ApoA is entirely novel [5,6,7].

The measurement of serum TC represents the total amount of cholesterol in the blood, which is mainly composed of LDL-C, HDL-C, and very low-density lipoprotein cholesterol [56]. LDL-C, commonly referred to as “bad cholesterol”, is associated with the initiation and progression of atherosclerosis [57,58]. The cumulative exposure of LDL-C over time increases the atherosclerotic plaque burden as more LDL particles accumulate in the artery wall; this buildup can lead to the obstruction of blood flow, resulting in CVD outcomes such as angina, myocardial infarction, and mortality [57]. In contrast, HDL-C, often referred to as “good cholesterol”, provides anti-atherosclerotic properties, such as the reverse transport of excess cholesterol from cells in the artery walls to the liver for removal, anti-inflammatory effects, and modulation of platelet aggregation [58,59,60,61]. Almond consumption is associated with significant reductions in LDL-C, with no effects on HDL-C, hence the observed reductions in other blood lipid outcomes, including non-HDL-C and a shift in the ratios of TC:HDL-C and LDL-C:HDL-C towards more protective cholesterol.

The effects of almond consumption on LDL particle size have been investigated in several studies [21,24,41,46,48]. Although Ruisinger et al. [48] reported a significant shift in LDL particle size from Pattern A (large, buoyant) to Pattern B (small, dense) in almond consumers versus non-consumers, this finding is not supported by the collective evidence from the other studies in which the effects of almond consumption on LDL particle size were assessed [21,24,41,46]. In all of these studies, almond consumption was associated with reductions in small, dense LDL particles [21,24,41,46], with the reductions reaching significance relative to the control in 2 of the studies [26,46]. Small, dense LDL particles are particularly atherogenic compared to larger LDL particles, given that small, dense LDL particles can more easily penetrate the endothelium by fitting through tiny gaps, remaining in circulation longer, and triggering inflammation and plaque formation, as they are more prone to oxidation. Subjects in whom almond consumption decreased small, dense LDL particles included those with elevated TC and LDL-C at baseline [21]; subjects who were overweight and obese with mildly elevated LDL-C [41]; subjects who were overweight and obese following energy-restricted diets [24]; and subjects with prediabetes [46]. Importantly, diets were rigidly controlled in the studies by Berryman et al. [21] and Lee et al. [41], as all meals and snacks were provided to all study participants. The reasons for the discrepant results for LDL particle size reported by Ruisinger et al. [48], who specifically studied individuals on a stable dose of statins, are unclear and require further investigation.

CVD risk is strongly influenced by lipid-related factors. Beyond traditional cholesterol measures (e.g., TC, LDL-C, HDL-C, and ratios of these), apolipoproteins are also important markers of risk, as they carry lipids in the bloodstream. ApoA1 is a structural and functional protein that constitutes up to 70% of high-density lipoprotein (HDL), often called “good cholesterol” [62]. ApoA1 is synthesized and secreted in the liver and plays a crucial role in reverse cholesterol transport, which is the process of removing excess cholesterol from peripheral tissues and delivering it back to the liver for excretion [63,64]. ApoA1 is also a cofactor for lecithin cholesterol acyl transferase, which helps esterify free cholesterol on HDL, allowing HDL to carry cholesterol more effectively in blood [62]. Through these functions, ApoA1-rich HDL particles can extract cholesterol from plaque-laden macrophages in arteries and therefore protect against atherosclerosis [65]. As higher ApoA1 levels correlate with higher HDL and better cholesterol clearance from arteries, it can be viewed as a proxy for HDL functionality. Additionally, ApoA1 has anti-inflammatory and antioxidant roles as part of HDL and helps maintain endothelial health [65]. In healthy adults, ApoA1 levels roughly range from 75 to 160 mg/dL in men and 80 to 175 mg/dL in women [66]. ApoA1 levels below ~75 mg/dL in men or ~80 mg/dL in women are considered low and possibly associated with higher risk of coronary artery disease [66]. Recent studies have even found that decreased serum ApoA1 levels are associated with poor survival [67]. In our meta-analysis, the impact of almond consumption on ApoA was assessed in 15 strata. There was no significant effect of almonds relative to a control on ApoA levels, not in the main analysis nor in any of the sensitivity analyses—a finding also corroborated in the systematic reviews and meta-analyses by Lee-Bravatti et al. [6] and Morvaridzadeh et al. [10]. Thus, it appears that almonds do not reduce TC and LDL-C levels via reverse cholesterol transport.

On the other hand, ApoB is a “bad cholesterol”, where its large form, ApoB-100, is the primary protein carrier of all major atherogenic lipoproteins found in blood, including LDL, very low-density lipoprotein, intermediate-density lipoprotein, and Lp(a). The other form, ApoB-48, is irrelevant, as it is primarily made in intestines for chylomicrons [68]. Each lipoprotein particle contains exactly one molecule of ApoB-100, meaning the plasma ApoB concentration reflects the number of circulating LDL (and other Apo-B-containing) particles. This is important because atherosclerosis risk closely relates to the number of LDL particles that can infiltrate the arterial wall (i.e., particle count) rather than just the total cholesterol amount [69]. LDL and related lipoproteins carrying ApoB can enter the subendothelial space, where they may be retained and oxidized, triggering inflammation and plaque formation [41]. Thus, ApoB drives the development of atherosclerosis by ferrying cholesterol into arterial plaques. In healthy adults, ApoB levels roughly range from 66 to 133 mg/dL in men and 60 to 117 mg/dL in women [70]. ApoB levels over ~133 mg/dL in men or ~117 mg/dL in women are considered elevated. Individuals with normal LDL-C levels can still have elevated ApoB if they have a large number of small LDL particles. Therefore, elevated ApoB is strongly associated with greater risk of coronary artery disease, even if LDL levels are normal. Indeed, multiple studies have reported that ApoB levels correlate with CVD risk more robustly than LDL-C levels. For example, a meta-analysis conducted by Sniderman et al. [71] on 233,455 subjects found that ApoB (relative risk reduction [RRR] = 1.43) was the strongest predictor of cardiovascular events when directly compared to LDL-C (RRR = 1.34). Based on our meta-analysis results, the consumption of almonds was associated with a significant reduction in ApoB; this was observed in the main meta-analysis involving all 15 strata in which effects on ApoB were assessed and in 12 of the 14 sensitivity analyses. Only in the sensitivity analyses restricted to studies that were parallel in design and that were 12 weeks or longer in duration were there no effects of almonds on ApoB levels; however, the parallel studies and the longer-duration studies were conducted primarily in subjects with LDL-C levels that were optimal at baseline, and it is well-established that the magnitude of lipid lowering is very much a function of baseline lipid level (discussed further below). By reducing ApoB levels, the consumption of almonds interferes with the development of atherosclerosis by reducing the ferrying of cholesterol into arterial plaques.

The ApoB:ApoA1 ratio is the balance of atherogenic and anti-atherogenic factors [72]. In simple terms, it is a measure of the balance between “bad” cholesterol vehicles (ApoB) and “good” cholesterol vehicles (ApoA1) in the blood. An elevated ApoB:ApoA1 ratio signals that either ApoB, which promotes plaque formation, is increasing, or that ApoA1, which promotes plaque clearance, is decreasing. Overall, it indicates an unfavorable lipid profile and higher CVD risk, whereas a low ratio is protective. This ratio has also been considered by many experts as a CVD risk indicator; in fact, this ratio has been found to be a powerful predictor of myocardial infarction risk [73]. Based on a large international case–control study (INTERHEART) spanning 52 countries, the ApoB:ApoA1 ratio was shown to be a better marker for heart attack risk than the traditional TC:HDL-C ratio [73]. Based on another study conducted in China, the ApoB:ApoA1 ratio was a more effective predictor of coronary heart disease risk in overweight and obese individuals than traditional lipid measures like LDL-C [74]. The ratio was significantly associated with coronary heart disease occurrence and showed the highest predictive value among lipid markers in the overweight group [74]. In our main analysis and in 8 of the 12 sensitivity analyses, there was a statistically significant or borderline significant reduction in the ratio of ApoB:ApoA. In no other systematic review and meta-analysis was the effect of almond consumption on the ratio of ApoB:ApoA investigated.

Lp(a) is a specialized lipoprotein particle closely resembling LDL, but with an extra protein attached. Structurally, an Lp(a) particle consists of an LDL core (containing ApoB-100), with ApoA covalently bound to ApoB. The ApoA protein is unique to Lp(a) and has a peculiar kringle domain structure similar to plasminogen, though ApoA itself is not enzymatically active. This structural difference gives Lp(a) proatherogenic, proinflammatory, and antifibrinolytic properties. Because of its unique properties, Lp(a) is recognized as an independent risk factor for CVD. Not only can it contribute to arterial plaque buildup, but it is also associated with calcific aortic valve stenosis, a condition where heart valves become hardened and narrowed [75]. Unfortunately, Lp(a) levels are over 90% genetically determined by the Lp(a) gene, which encodes for ApoA, and diet or lifestyle has minimal impact on Lp(a) levels [76]. Indeed, as expected, the consumption of almonds had no significant impact on Lp(a), neither in our main meta-analysis nor in any of the sensitivity analyses—a finding that is consistent with reports by Lee-Bravatti et al. [6] and Morvaridzadeh et al. [10].

Based on the sensitivity analyses, greater improvements in lipid levels were observed when the meta-analyses were limited to strata in which the baseline LDL-C level was not optimal, an observation that has been reported previously for many different functional foods and food ingredients, including flaxseed [77], soy protein [78], oat beta-glucans [79,80], plant sterols and stanols [81,82], green tea catechins [83], nuts, in general [84], and almonds, specifically [5]. Of note, most of the studies in which the baseline LDL-C was not optimal were <12 weeks in duration and were crossover in design; thus, sensitivity analyses in which the pooled effect is larger for studies with a duration <12 weeks or those that were crossover in design are likely reflective of studies in which the baseline LDL-C level was not optimal. Likewise, most of the higher-dose studies were conducted in subjects with healthy baseline LDL-C levels, thereby obscuring an assessment of the impact of almond dose on the magnitude of lipid modification in subjects whose baseline LDL-C level was not optimal. In each of the studies by Jenkins et al. [37] and Sabaté et al. [49], the effects of 2 different doses of almonds on blood lipid levels were assessed in subjects whose baseline LDL-C levels were not optimal. In both studies, there was evidence of a dose-response, such that greater improvements in blood lipid levels were achieved with greater intakes of almonds.

The statistically significant improvements in levels of LDL-C, TC, non-HDL-C, TC:HDL-C, LDL-C:HDL-C, ApoB, and ApoB:ApoA observed in the main analyses persisted in our sensitivity analysis when higher quality studies were pooled separately from lower quality studies, thereby increasing confidence in the certainty of the findings. However, the reduction in TG levels, which was borderline significant in the main analysis, was not significant when the meta-analysis was restricted to either higher quality or lower quality studies, so the effect of almond consumption on TG levels remains uncertain. Fasting TG levels are associated with “extraordinarily high” intraindividual variability [85]. Indeed, based on measurements taken on 3 occasions, Bookstein et al. [86] reported that day-to-day variability in TC, LDL-C, and HDL-C ranged between 5 and 10%, while that for TG was 20%. Likewise, based on 2 clinic visits, Jacobs and Barrett-Connor [87] reported coefficients of variation of approximately 8% for TC and 25% for TG. Based on 4 blood samples collected at 2-week intervals, the coefficient of biological variation ranged between 6 and 9% for TC, HDL-C, ApoA1, ApoB, and LDL-C, but it was 28% for TG [88]. It is likely that dedicated clinical studies that are appropriately powered to assess the effects of almond consumption specifically on TG levels are needed.

The mechanisms by which blood lipid levels are improved with the consumption of almonds have been reviewed previously [5,6,7]. Almonds contain the phytosterol β-sitosterol, which inhibits cholesterol absorption through the displacement of dietary cholesterol from bile salt micelles [89]. Moreover, oleic acid and linoleic acid, the most abundant unsaturated fatty acids in almonds, have been shown to favorably improve serum fatty acid profiles [90,91,92]. Given the low SFA content of almonds, increased almond consumption could result in decreased intakes of potentially atherogenic SFA from the overall diet. Almonds have also been shown to significantly reduce body weight, waist circumference, and fat mass [93], and these favorable changes may contribute to improvements in blood lipid levels. Finally, almonds are an excellent source of vitamin E, which plays an important role in the protection of lipids from oxidative damage [94,95].

The results reported herein are consistent with those reported in previous systematic reviews and meta-analyses on the lipid-lowering effects of almond consumption [5,6,7]. Of note, the beneficial effects of almond consumption on the ratio of ApoB:ApoA are entirely novel and highly encouraging. Beyond improvements in blood lipid levels, almonds are nutrient-dense, and their consumption is associated with improvements in several other cardiometabolic risk factors, including body weight and waist circumference [93], endothelial function [30], blood pressure [96], markers of inflammation (e.g., C-reactive protein and interleukin-6) [97], and markers of oxidative stress (e.g., malondialdehyde) [98]. CVD is a complex disease precipitated not only by the deposition of atherogenic lipids into vessel walls and the formation of atherosclerotic plaque but also oxidation and inflammation [99,100]. Indeed, in prospective observational studies, the consumption of nuts, including almonds, was associated with reduced risks of CVD incidence and mortality [101,102]. Additionally, in the PREDIMED randomized controlled trial, a Mediterranean diet supplemented with either nuts (including almonds) or olive oil was associated with significant reductions in the risks of CVD events and mortality compared to a control diet low in fat [103].

Our systematic review and meta-analysis has many strengths, including the conducting of the literature search and data extraction in duplicate (i.e., by 2 individuals, independently of one another), the assessments of heterogeneity and publication bias, and the conducting of several sensitivity analyses. We did not search grey literature (such as clinical trial registries) or for dissertations and theses, nor did we utilize quantitative methods to address potential bias arising from selective reporting—2 notable limitations. Also, in all of our included studies, it was not possible to blind subjects and investigators, given that the investigational product was almonds; the degree to which performance bias impacts subject/investigator behaviors and ultimately the results of studies of whole foods on blood lipid levels is unknown. Likewise, although many of the identified studies were funded by industry, the risk of industry bias is low, as the cardioprotective value of nuts, including almonds, has been corroborated in prospective observational studies and even a randomized controlled trial (the PREDIMED study), which were funded predominantly by government agencies, not industry [101,102,103]. Finally, because this systematic review and meta-analysis builds on a systematic review and meta-analysis published almost a decade ago by Musa-Veloso et al. [5], some of the study data had already been extracted, and the work could not be prospectively registered on PROSPERO.

## 5. Conclusions

Almond consumption is associated with significant reductions in blood lipid levels, including LDL-C (−0.132 mmol/L; *p* < 0.001), TC (−0.160 mmol/L; *p* < 0.001), non-HDL-C (-0.204 mmol/L; *p* < 0.001), TC:HDL-C (−0.154; *p* = 0.001), and LDL-C:HDL-C (−0.112; *p* = 0.011), and a borderline significant reduction in TG (−0.037 mmol/L; *p* = 0.085), with no effects on HDL-C. ApoA1 is not significantly impacted by almond consumption (–0.714 mg/dL; *p* = 0.508); thus, reverse control transport is not a mechanism by which almonds improve lipid levels. In contrast, ApoB is significantly reduced with the consumption of almonds (−4.552 mg/dL, *p* < 0.001), as is the ApoB:ApoA1 ratio (–0.027 mg/dL, *p* = 0.006), implying that almonds reduce the ferrying of cholesterol into arterial plaques, which could reduce the risk of CVD outcomes. Unsurprisingly, Lp(a) levels are not significantly impacted by the consumption of almonds, likely because these levels are largely genetically determined and unlikely to change with dietary interventions. For lipid outcomes that were significantly improved with the consumption of almonds, the magnitude of the effect was larger when the analyses were limited to studies in which the baseline LDL-C level was not optimal. Almond consumption improves blood lipid levels, especially in those in whom LDL-C levels are not optimal, thereby potentially improving cardiovascular health and reducing the risk of CVD.

## Figures and Tables

**Table 1:** Overview of studies in which the effects of almonds on blood lipids were assessed (*n* = 36 publications and 48 strata).

Reference	Design ^a^ (Quality)	Duration (wk) ^b^	Country	Population ^c^		Investigational Product			Baseline Demographics ^d^			Background **Diet ^e^**
				*n* (Gender)	Health Status	Almonds (*n*)	Control (*n*)	Intended Matching	Age (y)	BMI (kg/m^2^)	LDL-C Status ^f^	
Abazarfard et al. [20]	P LQ	12	Iran	100F	BMI ≥ 25 kg/m^2^	50 g/d (*n* = 50)	NFD (*n* = 50)	CHO, E, PRO, TF	42.7 ± 7.1	29.6 ± 1.5	N.O.	LCD
Berryman et al. [21]	X HQ	6	U.S.	48 (22M, 26F)	OW; elevated TC and LDL-C	42.5 g/d	106 g/d muffin + 2.7 g/d butter	E, SFA	49.9 ± 9.4	26.2 ± 2.8	N.O.	BW maintenance diets
Bowen et al. [22]	SB, P HQ	8	Australia	76 (45M, 31F)	OW or OB; T2D or elevated FBG	56 g/d (*n* = 39)	72 g/d biscuits (*n* = 37)	E	60.7 ± 7.7	33.8 ± 5.6	O	Habitual diet
Brown et al. [23]	SB, P HQ	*52*	New Zealand	109 (GD NR)	Normal/ OW	42.5 g/d (*n* = 57)	Cookies + crackers (*n* = 52)	E	35.6 ± 13.4	23.7 ± 3.0	O	Usual snacks replaced
Carter et al. [24] ^g^	SB, P HQ	36	Australia	106 (GD NR)	OW or OB, with HTN and HC in some subjects	30 to 50 g/d (*n* = 55)	Cereal bar + rice crackers (*n* = 51)	E	47.5 ± *10.9*	30.7 ± *0.8*	O	LCD (12 wk); BW maintenance (24 wk)
Chen et al. [25]	X HQ	12	Taiwan	33 (13M, 20F)	T2D	60 g/d	NFD	E, PRO, PUFA, SFA	54.9 ± 10.5	25.5 ± 4.2	O	NCEP Step II BW maintenance diet
Coates et al. [26] ^g^	SB, P HQ	12	Australia	128 (GD NR)	OW/OB, with HTN and HC in some subjects	*~57* g/d (*n* = 63)	Biscuits + potato chips (*n* = 65)	E	65 ± 8	30.4 ± 3.7	O	Habitual diet
Cohen and Johnston [27] ^g^	P HQ	12	U.S.	13 (7M, 6F)	T2D	28 g/d (*n* = 6)	Two sticks of cheese (*n* = 7)	E, TF, CHO	66 ± 8.4	34.8 ± 8.0	O	No instructions provided
Damasceno et al. [28]	SB, X HQ	4	Spain	18 (9M, 9F)	Moderately HC	50 to 75 g/d	35 to 50 g/d olive oil	E, TF	56 ± 13	25.7 ± 2.3	N.O.	Mediterranean diet
Dhillon et al. [29]	SB, P HQ	8	U.S.	73 (32M, 41F)	Low/ moderate cardio-metabolic risk	56.7 g/d (*n* = 38)	77.5 g/d graham crackers (*n* = 35)	E	18.0 ± NR	25.5 ± 4.7	O	No instructions provided
Dikaryanto et al. [30]	SB, P HQ	6	UK	102 (GD NR)	Above- average CVD risk	*56* g/d (*n* = 53)	Muffins (*n* = 49)	E	56.2 ± 10.4	27.0 ± 4.4	N.O.	Habitual diet
Foster et al. [31]	P LQ	*72*	U.S.	92 (GD NR)	OW	56 g/d + LCD (*n* = 47)	Nut-free LCD (*n* = 45)	N/A	46.8 ± 12.5	34.0 ± 3.6	O	Habitual diet (1 wk) followed by LCD
Gayathri et al. [32]	P HQ	12	India	352 (GD NR)	OW and OB; elevated CMD risk factors	43 g/d (*n* = 174)	NFD (n = 178)	N/A	37.7 ± 8.6	28.5 ± 3.8	O	Habitual diet
Gravesteijn et al. [33]	X LQ	21	NL	34 (22M, 12F)	OW and OB; prediabetic	50 g/d	NFD	N/A	*63.7* ± *10.1*	*29.6* ± *5.3*	N.O.	Dutch dietary guidelines
Gulati et al. [34]	P HQ	~*13* (90 d)	India	60 (GD NR)	Prediabetic	60 g/d (*n* = 30)	NFD (*n* = 30)	N/A	42.0 ± 8.2	31 ± 4.2	O	Guidelines for Asian Indians
Huang et al. [35]	P HQ	16	U.S.	81 (23M, 58F)	Elevated HbA1c	56 g/d (*n* = 39)	*83.4* g/d pretzels (*n* = 42)	E	49.4 ± *12.6*	35.2 ± *7.7*	O	No guidance provided
Hunter et al. [36]	P HQ	24	U.S.	118 (GD NR)	OW or OB	*42.5* g/d (*n* = 59)	NFD (*n* = 59)	N/A	37.0 ± NR	33.5 ± NR	O	Habitual diet
Jenkins et al. [37] (st 1)	X LQ	4	Canada	27 (15M, 12F)	HC	37 ± 2 g/d	75 ± 3 g/d muffins	E, FIB, PRO, PUFA, SFA	64 ± 9	25.7 ± 3.0	N.O.	Low-fat therapeutic diet
Jenkins et al. [37] (st 2)						73 ± 3 g/d	147 ± 6 g/d muffins					
Jia et al. [38] (st 1)	P HQ	4	China	30M	Habitual smokers	84 g/d (*n* = 10)	NFD (*n* = 10)	NR	22.3 ± 1.8	NR	O	Army unit canteen
Jia et al. [38] (st 2)						168 g/d (*n* = 10)			22.1 ± 1.8			
Jung et al. [39]	X HQ	4	Republic of Korea	84 (11M, 73F)	OW/OB	56 g/d	70 g/d cookies	E	52.4 ± 0.6	25.4 ± 0.22	O	Habitual diet
Kurlandsky and Stote [40] (st 1)	P HQ	6	U.S.	47F	HC	60 g/d (*n* = 12)	NFD (*n* = 12)	N/A	46.6 ± 10.4	25.7 ± 3.8	O	Self-selected diet based on NCEP ATP III TLC diet guidelines
Kurlandsky and Stote [40] (st 2)						60 g/d + 41 g/d dark chocolate (*n* = 11)	41 g/d dark chocolate (*n* = 12)	N/A	41.1 ± 11.1	25.5 ± 3.8		
Lee et al. [41] (st 1)	SB, X LQ	4	U.S.	31 (18M, 13F)	OW and OB; mildly elevated LDL-C	42.5 g/d	Butter, cheese, and refined grains	E	46.3 ± *10.0*	29.6 ± *2.8*	N.O.	Typical American diet
Lee et al. [41] (st 2)						42.5 g/d + 18 g/d natural cocoa + 43 g/d dark chocolate	18 g/d cocoa powder + 43 g/d dark chocolate	E				
Li et al. [42]	X HQ	4	Taiwan	20 (9M, 11F)	T2D; HC	56 g/d	NFD	E, PRO, SFA	58 ± *8.9*	26.0 ± *3.1*	N.O.	BW maintenance NCEP Step II diet
Liu et al. [43] (st 1)	SB, P LQ	16	Republic of Korea	169 (77M, 92F)	Healthy	Pre-meal: 56 g/d (*n* = 58)	66 g/d cookies (*n* = 56)	E	26.33 ± 5.55	22.59 ± 3.04	O	Habitual diet
Liu et al. [43] (st 2)						Snack: 56 g/d (n = 55)						
Lovejoy et al. [44] (st 1)	DB, X HQ	4	U.S.	30 (13M, 17F)	T2D	High fat: 57 to 113 g/d, depending on EER (37% fat, 10% from almonds)	High fat: olive or canola oil (37% fat, 10% from MUFAs in oil)	CHO, E, MUFA, PRO, PUFA, SFA, TF	53.8 ± *10.4*	33.0 ± *5.47*	O	BW maintenance diets
Lovejoy et al. [44] (st 2)						Low fat: 57 to 113 g/d, depending on EER (25% fat, 10% from almonds)	Low fat: olive or canola oil (25% fat, 10% from MUFAs in oil)	CHO, E, MUFA, PRO, PUFA, SFA, TF				
Mustra Rakic et al. [45] (st 1)	SB, P HQ	24	U.S.	60 (33M, 27F)	OW and OB	42 g/d (*n* = 19)	100 g/d snack mix (*n* = 17)	E, PRO, TF	62.3 ± 5.9	29.0 ± 2.7	O	BW maintenance diets
Mustra Rakic et al. [45] (st 2)						84 g/d (*n* = 24)		CHO	61.4 ± 6.4	29.0 ± 2.7		
Palacios et al. [46]	X LQ	6	U.S.	33 (17M, 16F)	OW and OB; prediabetes	85 g/d	CHO-based foods	E	48.3 ± *12.64*	30.5 ± *4.02*	O	Habitual diet
Rayo et al. [47]	X HQ	8	U.S.	26 (GD NR)	OW	57 g/d	86.5 g/d isocaloric pretzels	E	37.2 ± 6.3	26.2 ± 3.1	O	Habitual diet
Ruisinger et al. [48]	P HQ	4	U.S.	48 (24M, 24F)	On stable statin therapy	100 g/d (*n* = 22)	NFD (*n* = 26)	N/A	59.6 ± 11.0	29.2 ± 4.3	O	ATP-III TLC diet
Sabaté et al. [49] (st 1)	X HQ	4	U.S.	25 (14M, 11F)	Mildly HC	34 g/d	NFD	E, PRO	41 ± 13	NR	N.O.	Isoenergetic NCEP Step I diets
Sabaté et al. [49] (st 1)						68 g/d						
Siegel et al. [50]	X HQ	8	UK	25 (11M, 14F)	Mildly OW	57 g/d	86 g/d pretzels	E	35.1 ± 4.7	25.8 ± 3.6	O	Habitual diet
Spiller et al. [51] (st 1)	P HQ	4	U.S.	45 (12M, 33F)	HC	100 g/d (*n* = 18)	48 g olive oil + 113 g cottage cheese + 21 g rye crackers/d (*n* = 15)	E, TF, PRO	53 ± 10	NR	N.O.	Some foods provided for participants to prepare (e.g., lentils)
Spiller et al. [51] (st 2)							85 g cheddar cheese + 28 g butter + 21 g rye crackers/d (*n* = 12)	E, TF, PRO				
Sweazea et al. [52] ^g^	P HQ	12	U.S.	21 (9M, 12F)	T2D	43 g/d (*n* = 10)	NFD (*n* = 11)	N/A	56.2 ± 7.5	35.3 ± 8.3	O	Habitual diet
Tamizifar et al. [53]	SB, X HQ	4	Iran	30 (17M, 13F)	Mildly HC	25 g/d	NFD	E, CHO, PRO	56 ± 6.1	24.1 ± 4.5	N.O.	NCEP Step I diet
Tan and Mattes [54] (st 1)	P HQ	4	Australia	137 (48M, 89F)	OW or OB; increased T2D risk	Breakfast: 43 g/d (*n* = 28)	NFD (*n* = 27)	N/A	30.8 ± 10.6	27.6 ± 4.6	O	Habitual diet
Tan and Mattes [54] (st 2)						Morning snack: 43 g/d (*n* = 28)			28.2 ± 10.2	27.9 ± 4.7		
Tan and Mattes [54] (st 3)						Lunch: 43 g/d (*n* = 26)			29.0 ± 11.7	28.0 ± 4.2		
Tan and Mattes [54] (st 4)						Afternoon snack: 43 g/d (*n* = 28)			28.9 ± 10.8	27.6 ± 4.8		
Wien et al. [55]	P HQ	16	U.S.	54 (GD NR)	Prediabetic	*60* g/d + ADA diet (*n* = 25)	NFD (*n* = 29)	E	53.5 ± 10.0	29.5 ± 5.0	O	ADA diet plan

ADA = American Diabetes Association; ATP = Adult Treatment Program; BMI = body mass index; BW = body weight; CHO = carbohydrate; CMD = cardiometabolic disease; CVD = cardiovascular disease; d = day; DB = double-blind; E = energy; EER = estimated energy requirement; F = females; FIB = fiber; FBG = fasting blood glucose; GD = gender distribution; HbA1c = glycated hemoglobin; HC = hypercholesterolemic; HQ = higher quality; HTN = hypertension; LCD = low-calorie diet; LDL-C = low-density lipoprotein cholesterol; LQ = lower quality; M = males; MUFA = monounsaturated fatty acids; *n* = number; N/A = not applicable; NCEP = National Cholesterol Education Program; NFD = nut-free diet; NL = Netherlands; N.O. = not optimal; NR = not reported; O = optimal; OB = obese; OW = overweight; P = parallel; PRO = protein; PUFA = polyunsaturated fatty acids; SB = single-blind; SD = standard deviation; SFA = saturated fatty acids; st = stratum; T2D = type 2 diabetes; TC = total cholesterol; TF = total fat; TLC = Therapeutic Lifestyle Changes; UK = United Kingdom; U.S. = United States; X = crossover; wk = week(s); y = years. Values that are italicized were converted by the authors. ^a^ All studies were randomized and controlled, as required by the study inclusion criteria. ^b^ Duration refers to supplementation period and for X studies is per arm. ^c^ Study population (*n*) represents the number of subjects analyzed. ^d^ For P studies, values are reported as pooled mean ± pooled SD. ^e^ Shaded cells indicate that all study meals and snacks were provided to participants. ^f^ A mean LDL-C level ≥ 3.36 mmol/L, categorized as borderline-high by NCEP [17], was considered not optimal (N.O.); otherwise, the baseline LDL C level was categorized as optimal (O). ^g^ For these studies, the indicated amount of investigational product was consumed 6 d/wk [24,26], 5 d/wk [27], or 5 to 7 d/wk [52].

**Table 2:** Effects of almonds on blood lipid levels: Meta-analysis results.

Outcome	WMD (95% CI)	*p*-Value	Heterogeneity		Publication Bias	
			*I*^2^ (%)	*p*-Value	Missing (L or R of WMD) ^a^	WMD (95% CI) ^b^
LDL-C (mmol/L) (*n* = 35 studies, 46 strata, 2455 subjects)	−0.132 (−0.190, −0.075)	**<0.001**	63.215	**<0.001**	5 (R)	−0.11 (−0.16, −0.05)
TC (mmol/L) (*n* = 36 studies, 48 strata, 2485 subjects)	−0.160 (−0.218, −0.101)	**<0.001**	31.123	**0.034**	3 (R)	−0.13 (−0.20, −0.07)
HDL-C (mmol/L) (*n* = 34 studies, 45 strata, 2442 subjects)	−0.002 (−0.020, 0.016)	0.815	32.936	**0.029**	0 (L)	N/A; no publication bias
Non-HDL-C (mmol/L) (*n* = 8 studies, 10 strata, 621 subjects)	−0.204 (−0.281, −0.127)	**<0.001**	0.000	0.901	1 (R)	−0.20 (−0.27, −0.12)
TC:HDL-C (*n* = 15 studies, 18 strata, 1050 subjects)	−0.154 (−0.246, −0.063)	**0.001**	38.129	0.061	5 (R)	−0.07 (−0.18, 0.03)
LDL-C:HDL-C (*n* = 8 studies, 11 strata, 285 subjects)	−0.112 (−0.199, −0.026)	**0.011**	0.000	0.936	2 (R)	−0.10 (−0.19, −0.02)
TG (mmol/L) (*n* = 36 studies, 48 strata, 2485 subjects)	−0.037 (−0.079, 0.005)	0.085	23.299	0.097	3 (R)	−0.02 (−0.07, 0.03)
ApoA (mg/dL) ^c^ (*n* = 12 studies, 15 strata, 886 subjects)	−0.714 (−2.830, 1.402)	0.508	0.000	0.965	2 (L)	−0.93 (−3.01, 1.15)
ApoB (mg/dL) (*n* = 12 studies, 15 strata, 886 subjects)	−4.552 (−6.460, −2.645)	**<0.001**	4.349	0.403	0 (R)	N/A; no publication bias
ApoB:ApoA ^d^ (*n* = 7 studies, 10 strata, 268 subjects)	−0.027 (−0.046, −0.008)	**0.006**	0.000	0.955	1 (R)	−0.03 (−0.05, −0.01)
Lp(a) (mg/dL) (*n* = 6 studies, 9 strata, 197 subjects)	0.563 (−0.366, 1.492)	0.235	0.000	0.996	4 (R)	0.69 (−0.19, 1.57)

Apo = apolipoprotein; CI = confidence interval; HDL-C = high-density lipoprotein cholesterol; L = left; LDL-C = low-density lipoprotein cholesterol; Lp(a) = lipoprotein A; *n* = number; N/A = not applicable; R = right; TC = total cholesterol; TG = triglycerides; WMD = weighted mean difference. Bolded values indicate statistical significance (*p* < 0.05). ^a^ Denotes the number of strata found to be missing either to the right or left of the WMD, using the Trim and Fill method of Duval and Tweedie [19]. ^b^ The presented WMD is calculated with the imputation of the studies found to be missing according to the Trim and Fill method of Duval and Tweedie [19]). ^c^ For the studies that reported the results for ApoA1, the results for this outcome were combined with ApoA in the meta-analysis. ^d^ For the studies that reported the results for the ratio of ApoB:ApoA1, the results for this outcome were combined with the results for the ratio of ApoB:ApoA in the meta-analysis.

## Data Availability

The data used in the meta-analyses can be provided upon request. The listing of excluded studies also can be provided upon request.

## Supplementary Materials

The following supporting information can be downloaded at: https://www.mdpi.com/article/10.3390/nu17172791/s1: Supplementary Table S1: PRISMA 2020 Checklist; Supplementary Table S2: Summary of study quality using Health Canada’s Quality Appraisal Tool; Supplementary Table S3: Summary of outcomes reported across the studies; Supplementary Table S4: Main and sensitivity analysis results.

## Abbreviations

The following abbreviations are used in this manuscript:

ApoA	apolipoprotein A
ApoB	apolipoprotein B
CI	confidence interval
CVD	cardiovascular disease
GDN	Global Diagnostics Network
HDL	high-density lipoprotein
HDL-C	high-density lipoprotein cholesterol
ITT	intention-to-treat
LDL	low-density lipoprotein
LDL-C	low-density lipoprotein cholesterol
Lp(a)	lipoprotein a
MUFA	monounsaturated fat
PRIMSA	Preferred Reporting Items for Systematic Reviews and Meta-analyses
PUFA	polyunsaturated fat
RRR	relative risk reduction
SFA	saturated fat
T2D	type 2 diabetes
TC	total cholesterol
TG	Triglycerides
WHO	World Health Organization
