# Supplementary material for: Blood Lipid Levels in Response to Almond Consumption: A Systematic Review and Meta-Analysis of Randomized Controlled Trials
Source: Nutrients. 2025 Aug 28;17(17):2791. doi: 10.3390/nu17172791 (PMC12430622; doi:10.3390/nu17172791)
Supplement: Supplementary file 1 [file nutrients-17-02791-s001.zip › Supplementary Table 2 - Study Quality - 27Aug2025.pdf]

Supplementary Table 2. Summary of study quality using Health Canada's Quality Appraisal Tool.

Reference	Quality Score	Quality Rank (High or Low)[a]	Information Gaps[b]
Abazarfard et al. [20]	8/15	Low	• Randomization method NR and CD its appropriateness<br>• Allocation concealment NR<br>• Blinding of subjects and outcome assessors NR<br>• No results of an ITT analysis<br>• **Potential confounders were not considered**[c]
Berryman et al. [21]	11/15	High	• Allocation concealment NR<br>• Blinding of subjects and outcome assessors NR<br>• No results of an ITT analysis
Bowen et al. [22]	12/15	High	• Allocation concealment NR<br>• Blinding of subjects NR<br>• No results of an ITT analysis
Brown et al. [23]	14/15	High	• Blinding of subjects NR
Carter et al. [24]	13/15	High	• Allocation concealment NR<br>• Blinding of subjects NR
Chen et al. [25]	11/15	High	• Allocation concealment NR<br>• Blinding of subjects and outcome assessors NR<br>• No results of an ITT analysis
Coates et al. [26]	12/15	High	• Randomization method NR and CD its appropriateness<br>• Blinding of subjects NR
Cohen and Johnston [27]	9/15	High	• Randomization method NR and CD its appropriateness<br>• Allocation concealment NR<br>• Blinding of subjects and outcome assessors NR<br>• Inclusion/exclusion criteria NR
Damasceno et al. [28]	13/15	High	• Blinding of subjects NR<br>• No results of an ITT analysis
Dhillon et al. [29]	11/15	High	• Allocation concealment NR<br>• Blinding of subjects NR<br>• No results of an ITT analysis<br>• Reasons for attrition NR

Supplementary Table 2. Summary of study quality using Health Canada's Quality Appraisal Tool.

Reference	Quality Score	Quality Rank (High or Low)[a]	Information Gaps[b]
Dikaryanto et al. [30]	12/15	High	• Allocation concealment NR<br>• Blinding of subjects NR<br>• No results of an ITT analysis
Foster et al. [31]	11/15	Low	• Allocation concealment NR<br>• Blinding of subjects and outcome assessors NR<br>• **Potential confounders were not considered**[c]
Gayathri et al. [32]	11/15	High	• Allocation concealment NR<br>• Blinding of subjects and outcome assessors NR<br>• No results of an ITT analysis
Gravesteijn et al. [33]	11/15	Low	• Allocation concealment NR<br>• Blinding of subjects and outcome assessors NR<br>• **High attrition rate and no results of an ITT analysis**[d]
Gulati et al. [34]	12/15	High	• Blinding of subjects and outcome assessors NR<br>• Methodology used to measure the health effect NR
Huang et al. [35]	12/15	High	• Allocation concealment NR<br>• Blinding of subjects and outcome assessors NR
Hunter et al. [36]	12/15	High	• Blinding of subjects and outcome assessors NR<br>• Allocation concealment NR
Jenkins et al. [37 strata 1 and 2]	8/15	Low	• Inclusion/exclusion criteria NR<br>• Randomization method NR and CD its appropriateness<br>• Allocation concealment NR<br>• Blinding of subjects and outcome assessors NR<br>• **High attrition rate and no results of an ITT analysis**[d]
Jia et al. [38 strata 1 and 2]	10/15	High	• Randomization method NR and CD its appropriateness<br>• Allocation concealment NR<br>• Blinding of subjects and outcome assessors NR
Jung et al. [39]	11/15	High	• Allocation concealment NR<br>• Blinding of subjects and outcome assessors NR<br>• No results of an ITT analysis

Supplementary Table 2. Summary of study quality using Health Canada's Quality Appraisal Tool.

Reference	Quality Score	Quality Rank (High or Low)[a]	Information Gaps[b]
Kurlandsky and Stote [40 strata 1 and 2]	9/15	High	• Randomization method NR and CD its appropriateness<br>• Allocation concealment NR<br>• Blinding of subjects and outcome assessors NR<br>• No results of an ITT analysis
Lee et al. [41 strata 1 and 2]	12/15	Low	• Allocation concealment NR<br>• Blinding of subjects NR<br>• **High attrition rate and no results of an ITT analysis[d]**
Li et al. [42]	9/15	High	• Randomization method NR and CD its appropriateness<br>• Allocation concealment NR<br>• Blinding of subjects and outcome assessors NR<br>• No results of an ITT analysis
Liu et al. [43 strata 1 and 2]	11/15	Low	• Allocation concealment NR<br>• Blinding of subjects NR<br>• **High attrition rate and no results of an ITT analysis[d]**<br>• **Potential confounders were not considered[c]**
Lovejoy et al. [44 strata 1 and 2]	10/15	High	• Randomization method NR and CD its appropriateness<br>• Allocation concealment NR<br>• Blinding of subjects NR<br>• No results of an ITT analysis
Mustra Rakic et al. [45 strata 1 and 2]	12/15	High	• Allocation concealment NR<br>• Blinding of subjects NR<br>• No results of an ITT analysis
Palacios et al. [46]	9/15	Low	• Randomization method NR and CD its appropriateness<br>• Allocation concealment NR<br>• Blinding of subjects and outcome assessors NR<br>• **High attrition rate and no results of an ITT analysis[d]**
Rayo et al. [47]	11/15	High	• Allocation concealment NR<br>• Blinding of subjects and outcome assessors NR<br>• No results of an ITT analysis

Supplementary Table 2. Summary of study quality using Health Canada's Quality Appraisal Tool.

Reference	Quality Score	Quality Rank (High or Low)[a]	Information Gaps[b]
Ruisinger et al. [48]	9/15	High	• Randomization method NR and CD its appropriateness<br>• Allocation concealment NR<br>• Blinding of subjects and outcome assessors NR<br>• No results of an ITT analysis
Sabaté et al. [49 strata 1 and 2]	9/15	High	• Randomization method NR and CD its appropriateness<br>• Allocation concealment NR<br>• Blinding of subjects and outcome assessors NR<br>• No results of an ITT analysis
Siegel et al. [50]	9/15	High	• Randomization method NR and CD its appropriateness<br>• Allocation concealment NR<br>• Blinding of subjects and outcome assessors NR<br>• No results of an ITT analysis
Spiller et al. [51 strata 1 and 2]	8/15	High	• Inclusion/exclusion criteria NR<br>• Randomization method NR and CD its appropriateness<br>• Allocation concealment NR<br>• Blinding of subjects and outcome assessors NR<br>• No results of an ITT analysis
Sweazea et al. [52]	9/15	High	• Randomization method NR and CD its appropriateness<br>• Allocation concealment NR<br>• Blinding of subjects and outcome assessors NR<br>• No results of an ITT analysis
Tamizifar et al. [53]	10/15	High	• Randomization method NR and CD its appropriateness<br>• Allocation concealment NR<br>• Blinding of subjects NR<br>• No results of an ITT analysis
Tan and Mattes [54 strata 1 through 4]	10/15	High	• Allocation concealment NR<br>• Blinding of subjects and outcome assessors NR<br>• Methodology used to measure the health effect NR[e]<br>• No results of an ITT analysis

Supplementary Table 2. Summary of study quality using Health Canada's Quality Appraisal Tool.

Reference	Quality Score	Quality Rank (High or Low)[a]	Information Gaps[b]
Wien et al. [55]	13/15	High	• Blinding of subjects and outcome assessors NR

BL = baseline; CD = could not determine; ITT = intention-to-treat; LDL-C = low-density lipoprotein cholesterol; NR = not reported.

[a] Studies were rated as "higher quality" if a score of ≥8 out of 15 was achieved, or as "lower quality" if a score of ≤7 out of 15 was achieved. A study with a "fatal flaw" (i.e., high attrition rate and no ITT analysis, lack of dietary intake assessment, and significant differences at baseline in LDL-C levels or confounding dietary variables without statistical adjustments), regardless of the score, was noted to be of "lower quality."
[b] "Fatal flaws" are bolded.
[c] In these studies, dietary intakes either were NR or there were significant differences between groups in baseline nutrient intakes or baseline LDL-C levels, without appropriate statistical adjustments.
[d] Studies with an overall attrition rate of >20% are considered to have a high risk of bias if an ITT analysis was not conducted.
[e] A description of the methodology includes a description of the laboratory instruments (e.g., enzymatic assays, autoanalyzer) used to measure blood lipid concentrations.
