# Supplementary material for: Blood Lipid Levels in Response to Almond Consumption: A Systematic Review and Meta-Analysis of Randomized Controlled Trials
Source: Nutrients. 2025 Aug 28;17(17):2791. doi: 10.3390/nu17172791 (PMC12430622; doi:10.3390/nu17172791)
Supplement: Supplementary file 1 [file nutrients-17-02791-s001.zip › Supplementary Table 3 - Summary of Outcomes - 27Aug2025.pdf]

Supplementary Table 3. Summary of outcomes reported across the studies.

Reference	LDL-C	TC	HDL-C	Non-HDL-C	TC: HDL-C	LDL-C: HDL-C	TG	ApoA	ApoA1	ApoB	ApoB: ApoA	ApoB: ApoA1	Lp(a)
Abazarfard et al. [20]	✓	✓	✓	-	✓	-	✓	-	-	-	-	-	-
Berryman et al. [21]	✓	✓	✓	✓	✓	✓	✓	-	✓	✓	-	✓	✓
Bowen et al. [22]	✓	✓	✓	-	✓	-	✓	-	-	-	-	-	-
Brown et al. [23]	✓	✓	✓	-	✓	-	✓	✓	-	✓	-	-	-
Carter et al. [24]	✓	✓	✓	✓	✓	-	✓	-	✓	✓	-	-	-
Chen et al. [25]	✓	✓	✓	-	-	✓	✓	-	✓	✓	-	✓[a]	-
Coates et al. [26]	✓	✓	✓	-	✓	-	✓	-	-	-	-	-	-
Cohen and Johnston [27]	✓	✓	-	-	-	-	✓	-	-	-	-	-	-
Damasceno et al. [28]	✓	✓	✓	-	-	✓	✓	-	✓	✓	-	-	✓
Dhillon et al. [29]	✓	✓	✓	-	-	-	✓	-	-	-	-	-	-
Dikaryanto et al. [30]	✓	✓	✓	✓	✓	-	✓	-	-	-	-	-	-
Foster et al. [31]	✓	✓	✓	-	✓	-	✓	-	-	-	-	-	-
Gayathri et al. [32]	✓	✓	✓	-	-	-	✓	✓	-	✓	-	-	-
Gravesteijn et al. [33]	✓	✓	✓	-	-	-	✓	-	-	-	-	-	-

Supplementary Table 3. Summary of outcomes reported across the studies.

Reference	LDL-C	TC	HDL-C	Non-HDL-C	TC: HDL-C	LDL-C: HDL-C	TG	ApoA	ApoA1	ApoB	ApoB: ApoA	ApoB: ApoA1	Lp(a)
Gulati et al. [34]	✓	✓	✓	-	-	-	✓	-	-	-	-	-	-
Huang et al. [35]	✓	✓	✓	-	-	-	✓	-	-	-	-	-	-
Hunter et al. [36]	✓	✓	✓	-	-	-	✓	-	-	-	-	-	-
Jenkins et al. [37 stratum 1]	✓	✓	✓	-	✓	✓	✓	-	✓	✓	-	✓	✓
Jenkins et al. [37 stratum 2]	✓	✓	✓	-	✓	✓	✓	-	✓	✓	-	✓	✓
Jia et al. [38 stratum 1]	-	✓	-	-	-	-	✓	-	-	-	-	-	-
Jia et al. [38 stratum 2]	-	✓	-	-	-	-	✓	-	-	-	-	-	-
Jung et al. [39]	✓	✓	✓	✓	✓	✓	✓	✓	-	✓	✓	-	-
Kurlandsky and Stote [40 stratum 1]	✓	✓	✓	-	-	-	✓	-	-	-	-	-	-
Kurlandsky and Stote [40 stratum 2]	✓	✓	✓	-	-	-	✓	-	-	-	-	-	-
Lee et al. [41 stratum 1]	✓	✓	✓	✓	✓	-	✓	-	✓	✓	-	✓	✓
Lee et al. [41 stratum 2]	✓	✓	✓	✓	✓	-	✓	-	✓	✓	-	✓	✓
Li et al. [42]	✓	✓	✓	-	-	✓	✓	-	✓	✓	-	✓	-

Supplementary Table 3. Summary of outcomes reported across the studies.

Reference	LDL-C	TC	HDL-C	Non-HDL-C	TC: HDL-C	LDL-C: HDL-C	TG	ApoA	ApoA1	ApoB	ApoB: ApoA	ApoB: ApoA1	Lp(a)
Liu et al. [43 stratum 1]	✓	✓	✓	✓	-	-	✓	-	-	-	-	-	-
Liu et al. [43 stratum 2]	✓	✓	✓	✓	-	-	✓	-	-	-	-	-	-
Lovejoy et al. [44 stratum 1]	✓	✓	✓	-	✓	✓	✓	-	-	-	-	-	-
Lovejoy et al. [44 stratum 2]	✓	✓	✓	-	✓	✓	✓	-	-	-	-	-	-
Mustra Rakic et al. [45 stratum 1]	✓	✓	✓	-	-	-	✓	-	-	-	-	-	-
Mustra Rakic et al. [45 stratum 2]	✓	✓	✓	-	-	-	✓	-	-	-	-	-	-
Palacios et al. [46]	✓	✓	✓	✓	✓	-	✓	-	✓	✓	-	-	-
Rayo et al. [47]	✓	✓	✓	-	-	-	✓	-	-	-	-	-	-
Ruisinger et al. [48]	✓	✓	✓	✓	-	-	✓	-	-	-	-	-	✓
Sabaté et al. [49 stratum 1]	✓	✓	✓	-	-	✓	✓	✓	-	✓	✓	-	✓
Sabaté et al. [49 stratum 2]	✓	✓	✓	-	-	✓	✓	✓	-	✓	✓	-	✓
Siegel et al. [50]	✓	✓	✓	-	-	-	✓	-	-	-	-	-	-
Spiller et al. [51 stratum 1]	✓	✓	✓	-	-[b]	-	✓	-	-	-	-	-	-

Supplementary Table 3. Summary of outcomes reported across the studies.

Reference	LDL-C	TC	HDL-C	Non-HDL-C	TC: HDL-C	LDL-C: HDL-C	TG	ApoA	ApoA1	ApoB	ApoB: ApoA	ApoB: ApoA1	Lp(a)
Spiller et al. [51 stratum 2]	✓	✓	✓	-	-[b]	-	✓	-	-	-	-	-	-
Sweazea et al. [52]	✓	✓	✓	-	-	-	✓	-	-	-	-	-	-
Tamizifar et al. [53]	✓	✓	✓	-	✓	-	✓	-	-	-	-	-	-
Tan and Mattes [54 stratum 1]	✓	✓	✓	-	-	-	✓	-	-	-	-	-	-
Tan and Mattes [54 stratum 2]	✓	✓	✓	-	-	-	✓	-	-	-	-	-	-
Tan and Mattes [54 stratum 3]	✓	✓	✓	-	-	-	✓	-	-	-	-	-	-
Tan and Mattes [54 stratum 4]	✓	✓	✓	-	-	-	✓	-	-	-	-	-	-
Wien et al. [55]	✓	✓	✓	-	✓	-	✓	-	-	-	-	-	-
**Total Strata in Meta-Analysis**	**46**	**48**	**45**	**10**	**18**	**11**	**48**	**5**[c]	**10**[c]	**15**	**3**[d]	**7**[d]	**9**

ApoA = apolipoprotein A; ApoA1 = apolipoprotein A1; ApoB = apolipoprotein B; HDL-C = high-density lipoprotein cholesterol; LDL-C = low-density lipoprotein cholesterol; Lp(a) = lipoprotein A; TC = total cholesterol; TG = triglycerides.

[a] Although Chen et al. [25] reported that the ratio of ApoA1:ApoB was evaluated, based on the values, it was assumed that this was a typo in the publication, and the values represented the ratio of ApoB:ApoA1.
[b] Although this outcome was said to have been assessed as part of the study, quantitative results (either numerical data or in figure form) were not reported for this outcome; therefore, the results could not be included in the meta-analysis.
[c] For the studies in which the results for ApoA1 were reported, the results were combined with ApoA in the meta-analysis.
[d] For the studies in which the results for the ratio of ApoB:ApoA1 were reported, the results were combined with the ratio of ApoB:ApoA in the meta-analysis.
