# Supplementary material for: Blood Lipid Levels in Response to Almond Consumption: A Systematic Review and Meta-Analysis of Randomized Controlled Trials
Source: Nutrients. 2025 Aug 28;17(17):2791. doi: 10.3390/nu17172791 (PMC12430622; doi:10.3390/nu17172791)
Supplement: Supplementary file 1 [file nutrients-17-02791-s001.zip › Supplementary Table 4 - Table of Meta-Analysis Results - 27Aug2025.pdf]

Supplementary Table 4. Main and sensitivity analysis results.
Sensitivity analyses were conducted for outcomes that were evaluated in at least 2 strata.

		LDL-C (mmol/L)	TC (mmol/L)	HDL-C (mmol/L)	Non-HDL-C (mmol/L)	TC:HDL-C	LDL-C:HDL-C	TG (mmol/L)	ApoA (mg/dL)[a]	ApoB (mg/dL)	ApoB:ApoA[b]	Lp(a) (mg/dL)
**All Strata**		$n = 46$	$n = 48$	$n = 45$	$n = 10$	$n = 18$	$n = 11$	$n = 48$	$n = 15$	$n = 15$	$n = 10$	$n = 9$
		-0.132	-0.160	-0.002	-0.204	-0.154	-0.112	-0.037	-0.714	-4.552	-0.027	0.563
		(-0.190, -0.075)	(-0.218, -0.101)	(-0.020, 0.016)	(-0.281, -0.127)	(-0.246, -0.063)	(-0.199, -0.026)	(-0.079, 0.005)	(-2.830, 1.402)	(-6.460, -2.645)	(-0.046, -0.008)	(-0.366, 1.492)
		**$p < 0.001$**	**$p < 0.001$**	$p = 0.815$	**$p < 0.001$**	**$p = 0.001$**	**$p = 0.011$**	$p = 0.085$	$p = 0.508$	**$p < 0.001$**	**$p = 0.006$**	$p = 0.235$
**Almond Dose**	**<45 g/d**	$n = 17$	$n = 17$	$n = 16$	$n = 4$	$n = 7$	$n = 3$	$n = 17$	$n = 8$	$n = 8$	$n = 5$	$n = 5$
		-0.143	-0.149	-0.016	-0.223	-0.239	-0.174	-0.040	-0.573	-4.392	-0.039	0.685
		(-0.228, -0.058)	(-0.230, -0.067)	(-0.055, 0.023)	(-0.341, -0.105)	(-0.364, -0.114)	(-0.338, -0.011)	(-0.102, 0.022)	(-3.114, 1.968)	(-6.746, -2.037)	(-0.070, -0.008)	(-0.303, 1.673)
		**$p = 0.001$**	**$p < 0.001$**	$p = 0.420$	**$p < 0.001$**	**$p < 0.001$**	**$p = 0.036$**	$p = 0.210$	$p = 0.659$	**$p < 0.001$**	**$p = 0.014$**	$p = 0.174$
	**≥45 g/d**	$n = 29$	$n = 31$	$n = 29$	$n = 6$	$n = 11$	$n = 8$	$n = 31$	$n = 7$	$n = 7$	$n = 5$	$n = 4$
		-0.128	-0.172	0.005	-0.189	-0.123	-0.088	-0.038	-1.035	-5.418	-0.020	-0.368
		(-0.194, -0.061)	(-0.251, -0.093)	(-0.017, 0.028)	(-0.291, -0.088)	(-0.239, -0.006)	(-0.190, 0.014)	(-0.094, 0.018)	(-4.857, 2.787)	(-9.769, -1.066)	(-0.044, 0.005)	(-3.093, 2.358)
		**$p < 0.001$**	**$p < 0.001$**	$p = 0.655$	**$p < 0.001$**	**$p = 0.039$**	$p = 0.092$	$p = 0.181$	$p = 0.596$	**$p = 0.015$**	$p = 0.115$	$p = 0.792$
**Baseline Lipid Level**	**Not Optimal**	$n = 15$	$n = 15$	$n = 15$	$n = 4$	$n = 8$	$n = 7$	$n = 15$	$n = 9$	$n = 9$	$n = 8$	$n = 8$
		-0.229	-0.290	-0.028	-0.210	-0.291	-0.195	-0.076	-0.029	-5.776	-0.035	0.646
		(-0.358, -0.100)	(-0.409, -0.172)	(-0.060, 0.004)	(-0.320, -0.100)	(-0.497, -0.084)	(-0.330, -0.061)	(-0.210, 0.058)	(-2.992, 2.935)	(-8.510, -3.041)	(-0.061, -0.010)	(-0.332, 1.624)
		**$p < 0.001$**	**$p < 0.001$**	$p = 0.083$	**$p < 0.001$**	**$p = 0.006$**	**$p = 0.004$**	$p = 0.266$	$p = 0.985$	**$p < 0.001$**	**$p = 0.007$**	$p = 0.196$
	**Optimal**	$n = 31$	$n = 33$	$n = 30$	$n = 6$	$n = 10$	$n = 4$	$n = 33$	$n = 6$	$n = 6$	$n = 2$	N/A
		-0.094	-0.109	0.013	-0.197	-0.084	-0.054	-0.034	-1.428	-3.149	-0.016	
		(-0.139, -0.048)	(-0.161, -0.056)	(-0.012, 0.037)	(-0.305, -0.090)	(-0.171, 0.003)	(-0.167, 0.060)	(-0.071, 0.003)	(-4.450, 1.595)	(-5.946, -0.353)	(-0.046, 0.013)	
		**$p < 0.001$**	**$p < 0.001$**	$p = 0.307$	**$p < 0.001$**	$p = 0.057$	$p = 0.354$	$p = 0.075$	$p = 0.355$	**$p = 0.027$**	$p = 0.272$	

Supplementary Table 4. Main and sensitivity analysis results.
Sensitivity analyses were conducted for outcomes that were evaluated in at least 2 strata.

		**LDL-C (mmol/L)**	**TC (mmol/L)**	**HDL-C (mmol/L)**	**Non-HDL-C (mmol/L)**	**TC:HDL-C**	**LDL-C:HDL-C**	**TG (mmol/L)**	**ApoA (mg/dL)[a]**	**ApoB (mg/dL)**	**ApoB:ApoA[b]**	**Lp(a) (mg/dL)**
**Study Design**	**Cross-over**	*n* = 18<br>-0.179<br>(-0.243, -0.115)<br>***p* < 0.001**	*n* = 18<br>-0.167<br>(-0.240, -0.095)<br>***p* < 0.001**	*n* = 18<br>-0.025<br>(-0.052, 0.003)<br>*p* = 0.077	*n* = 5<br>-0.200<br>(-0.304, -0.095)<br>***p* < 0.001**	*n* = 10<br>-0.150<br>(-0.265, -0.034)<br>***p* = 0.011**	*n* = 11<br>-0.112<br>(-0.199, -0.026)<br>***p* = 0.011**	*n* = 18<br>0.001<br>(-0.056, 0.057)<br>*p* = 0.983	*n* = 12<br>-0.473<br>(-2.926, 1.980)<br>*p* = 0.706	*n* = 12<br>-4.757<br>(-6.916, -2.598)<br>***p* < 0.001**	*n* = 10<br>-0.027<br>(-0.046, -0.008)<br>***p* = 0.006**	*n* = 8<br>0.646<br>(-0.332, 1.624)<br>*p* = 0.196
	**Parallel**	*n* = 28<br>-0.090<br>(-0.156, -0.024)<br>***p* = 0.007**	*n* = 30<br>-0.165<br>(-0.256, -0.073)<br>***p* < 0.001**	*n* = 27<br>0.009<br>(-0.015, 0.033)<br>*p* = 0.457	*n* = 5<br>-0.208<br>(-0.322, -0.094)<br>***p* < 0.001**	*n* = 8<br>-0.164<br>(-0.313, -0.015)<br>***p* = 0.031**	N/A	*n* = 30<br>-0.067<br>(-0.127, -0.007)<br>***p* = 0.028**	*n* = 3<br>-1.417<br>(-5.601, 2.767)<br>*p* = 0.507	*n* = 3<br>-2.914<br>(-8.706, 2.878)<br>*p* = 0.324	N/A	N/A **Error! Bookmark not defined.**
**Study Duration**	**<12 wk**	*n* = 28<br>-0.156<br>(-0.222, -0.090)<br>***p* < 0.001**	*n* = 30<br>-0.144<br>(-0.211, -0.078)<br>***p* < 0.001**	*n* = 28<br>-0.004<br>(-0.033, 0.026)<br>*p* = 0.816	*n* = 7<br>-0.208<br>(-0.298, -0.117)<br>***p* < 0.001**	*n* = 12<br>-0.110<br>(-0.198, -0.022)<br>***p* = 0.015**	*n* = 10<br>-0.118<br>(-0.209, -0.026)<br>***p* = 0.012**	*n* = 30<br>-0.022<br>(-0.066, 0.023)<br>*p* = 0.338	*n* = 11<br>-0.540<br>(-3.112, 2.032)<br>*p* = 0.681	*n* = 11<br>-5.111<br>(-7.162, -3.060)<br>***p* < 0.001**	*n* = 9<br>-0.029<br>(-0.049, -0.009)<br>***p* = 0.004**	*n* = 9<br>0.563<br>(-0.366, 1.492)<br>*p* = 0.235
	**≥12 wk**	*n* = 18<br>-0.083<br>(-0.153, -0.013)<br>***p* = 0.020**	*n* = 18<br>-0.186<br>(-0.294, -0.077)<br>***p* = 0.001**	*n* = 17<br>0.001<br>(-0.023, 0.025)<br>*p* = 0.956	*n* = 3<br>-0.193<br>(-0.341, -0.045)<br>***p* = 0.011**	*n* = 6<br>-0.242<br>(-0.456, -0.027)<br>***p* = 0.027**	N/A **Error! Bookmark not defined.**	*n* = 18<br>-0.062<br>(-0.151, 0.027)<br>*p* = 0.174	*n* = 4<br>-1.079<br>(-4.801, 2.642)<br>*p* = 0.570	*n* = 4<br>-2.145<br>(-6.586, 2.296)<br>*p* = 0.344	N/A **Error! Bookmark not defined.**	N/A
**Lipid-Lowering Medication Use**	**None**	*n* = 34<br>-0.137<br>(-0.206, -0.068)<br>***p* < 0.001**	*n* = 36<br>-0.173<br>(-0.246, -0.100)<br>***p* < 0.001**	*n* = 34<br>-0.011<br>(-0.029, 0.008)<br>*p* = 0.265	*n* = 7<br>-0.203<br>(-0.288, -0.119)<br>***p* < 0.001**	*n* = 11<br>-0.172<br>(-0.321, -0.024)<br>***p* = 0.023**	*n* = 8<br>-0.113<br>(-0.208, -0.018)<br>***p* = 0.019**	*n* = 36<br>-0.027<br>(-0.078, 0.024)<br>*p* = 0.294	*n* = 10<br>-1.399<br>(-3.867, 1.069)<br>*p* = 0.267	*n* = 10<br>-4.965<br>(-7.844, -2.086)<br>***p* = 0.001**	*n* = 7<br>-0.028<br>(-0.050, -0.007)<br>***p* = 0.008**	*n* = 6<br>0.683<br>(-0.308, 1.675)<br>*p* = 0.177
	**Some or All**	*n* = 12<br>-0.132<br>(-0.219, -0.045)<br>***p* = 0.003**	*n* = 12<br>-0.137<br>(-0.234, -0.039)<br>***p* = 0.006**	*n* = 11<br>0.030<br>(-0.014, 0.074)<br>*p* = 0.184	*n* = 3<br>-0.203<br>(-0.398, -0.008)<br>***p* = 0.042**	*n* = 7<br>-0.144<br>(-0.260, -0.029)<br>***p* = 0.014**	*n* = 3<br>-0.108<br>(-0.320, 0.104)<br>*p* = 0.316	*n* = 12<br>-0.073<br>(-0.155, 0.009)<br>*p* = 0.079	*n* = 5<br>1.187<br>(-2.926, 5.300)<br>*p* = 0.572	*n* = 5<br>-3.846<br>(-7.203, -0.489)<br>***p* = 0.025**	*n* = 3<br>-0.021<br>(-0.067, 0.026)<br>*p* = 0.385	*n* = 3<br>-0.303<br>(-2.963, 2.358)<br>*p* = 0.823

Supplementary Table 4. Main and sensitivity analysis results.
Sensitivity analyses were conducted for outcomes that were evaluated in at least 2 strata.

		**LDL-C (mmol/L)**	**TC (mmol/L)**	**HDL-C (mmol/L)**	**Non-HDL-C (mmol/L)**	**TC:HDL-C**	**LDL-C:HDL-C**	**TG (mmol/L)**	**ApoA (mg/dL)[a]**	**ApoB (mg/dL)**	**ApoB:ApoA[b]**	**Lp(a) (mg/dL)**
**Study Quality**	**Lower**	$n = 10$<br>-0.101<br>(-0.209, 0.008)<br>$p = 0.068$	$n = 10$<br>-0.228<br>(-0.395, -0.061)<br>**$p = 0.007$**	$n = 10$<br>-0.008<br>(-0.014, -0.002)<br>**$p = 0.009$**	$n = 5$<br>-0.167<br>(-0.283, -0.051)<br>**$p = 0.005$**	$n = 7$<br>-0.270<br>(-0.464, -0.075)<br>**$p = 0.007$**	$n = 2$<br>-0.182<br>(-0.543, 0.180)<br>$p = 0.325$	$n = 10$<br>-0.118<br>(-0.275, 0.040)<br>$p = 0.143$	$n = 5$<br>-0.455<br>(-4.235, 3.326)<br>$p = 0.814$	$n = 5$<br>-4.393<br>(-7.713, -1.073)<br>**$p = 0.010$**	$n = 4$<br>-0.044<br>(-0.088, 0.001)<br>$p = 0.054$	$n = 4$<br>0.443<br>(-0.818, 1.705)<br>$p = 0.491$
	**Higher**	$n = 36$<br>-0.138<br>(-0.190, -0.086)<br>**$p < 0.001$**	$n = 38$<br>-0.140<br>(-0.197, -0.082)<br>**$p < 0.001$**	$n = 35$<br>0.001<br>(-0.025, 0.026)<br>$p = 0.952$	$n = 5$<br>-0.232<br>(-0.335, -0.130)<br>**$p < 0.001$**	$n = 11$<br>-0.088<br>(-0.171, -0.006)<br>**$p = 0.036$**	$n = 9$<br>-0.108<br>(-0.197, -0.019)<br>**$p = 0.018$**	$n = 38$<br>-0.026<br>(-0.063, 0.012)<br>$p = 0.179$	$n = 10$<br>-0.833<br>(-3.386, 1.721)<br>$p = 0.523$	$n = 10$<br>-4.611<br>(-7.589, -1.633)<br>**$p = 0.002$**	$n = 6$<br>-0.023<br>(-0.045, -0.002)<br>**$p = 0.032$**	$n = 5$<br>0.705<br>(-0.669, 2.078)<br>$p = 0.315$
**Meals or Snacks Provided**	**No**	$n = 37$<br>-0.124<br>(-0.187, -0.060)<br>**$p < 0.001$**	$n = 37$<br>-0.165<br>(-0.239, -0.091)<br>**$p < 0.001$**	$n = 36$<br>-0.001<br>(-0.022, 0.020)<br>$p = 0.918$	$n = 7$<br>-0.202<br>(-0.298, -0.107)<br>**$p < 0.001$**	$n = 13$<br>-0.162<br>(-0.272, -0.053)<br>**$p = 0.004$**	$n = 4$<br>-0.122<br>(-0.262, 0.018)<br>$p = 0.088$	$n = 37$<br>-0.042<br>(-0.091, 0.007)<br>$p = 0.095$	$n = 8$<br>-1.654<br>(-4.735, 1.428)<br>$p = 0.293$	$n = 8$<br>-4.186<br>(-6.737, -1.636)<br>**$p = 0.001$**	$n = 3$<br>-0.024<br>(-0.052, 0.005)<br>$p = 0.106$	$n = 4$<br>-0.347<br>(-2.972, 2.279)<br>$p = 0.796$
	**Yes**	$n = 9$<br>-0.164<br>(-0.243, -0.084)<br>**$p < 0.001$**	$n = 11$<br>-0.152<br>(-0.251, -0.054)<br>**$p = 0.003$**	$n = 9$<br>-0.005<br>(-0.050, 0.039)<br>$p = 0.811$	$n = 3$<br>-0.206<br>(-0.336, -0.076)<br>**$p = 0.002$**	$n = 5$<br>-0.140<br>(-0.345, 0.065)<br>$p = 0.180$	$n = 7$<br>-0.107<br>(-0.217, 0.004)<br>$p = 0.058$	$n = 11$<br>-0.011<br>(-0.123, 0.101)<br>$p = 0.848$	$n = 7$<br>0.124<br>(-2.787, 3.035)<br>$p = 0.933$	$n = 7$<br>-5.412<br>(-9.915, -0.908)<br>**$p = 0.019$**	$n = 7$<br>-0.030<br>(-0.056, -0.004)<br>**$p = 0.024$**	$n = 5$<br>0.693<br>(-0.300, 1.686)<br>$p = 0.171$

ApoA = apolipoprotein A; ApoA1 = apolipoprotein A1; ApoB = apolipoprotein B; HDL-C = high-density lipoprotein cholesterol; LDL-C = low-density lipoprotein cholesterol; Lp(a) = lipoprotein A; n = number; N/A = not applicable; TC = total cholesterol; TG = triglycerides.

[a] For the studies that reported the results for ApoA1, the results for this outcome were combined with ApoA in the meta-analysis.
[b] For the studies that reported the results for the ratios of ApoB:ApoA1, the results for this outcome were combined with ApoB:ApoA in the meta-analysis.
